# Evidence for Novel Hepaciviruses in Rodents

**DOI:** 10.1371/journal.ppat.1003438

**Published:** 2013-06-20

**Authors:** Jan Felix Drexler, Victor Max Corman, Marcel Alexander Müller, Alexander N. Lukashev, Anatoly Gmyl, Bruno Coutard, Alexander Adam, Daniel Ritz, Lonneke M. Leijten, Debby van Riel, Rene Kallies, Stefan M. Klose, Florian Gloza-Rausch, Tabea Binger, Augustina Annan, Yaw Adu-Sarkodie, Samuel Oppong, Mathieu Bourgarel, Daniel Rupp, Bernd Hoffmann, Mathias Schlegel, Beate M. Kümmerer, Detlev H. Krüger, Jonas Schmidt-Chanasit, Alvaro Aguilar Setién, Veronika M. Cottontail, Thiravat Hemachudha, Supaporn Wacharapluesadee, Klaus Osterrieder, Ralf Bartenschlager, Sonja Matthee, Martin Beer, Thijs Kuiken, Chantal Reusken, Eric M. Leroy, Rainer G. Ulrich, Christian Drosten

**Affiliations:** 1 Institute of Virology, University of Bonn Medical Centre, Bonn, Germany; 2 Chumakov Institute of Poliomyelitis and Viral Encephalitides, Moscow, Russia; 3 Lomonosov Moscow State University, Moscow, Russia; 4 Architectures et Fonctions des Macromolécules Biologiques, UMR 7257 CNRS and Aix-Marseille University, Marseille, France; 5 Institute of Pathology, University of Cologne Medical Centre, Cologne, Germany; 6 Erasmus MC, Department of Viroscience, Rotterdam, The Netherlands; 7 Institute of Experimental Ecology, University of Ulm, Ulm, Germany; 8 Noctalis, Centre for Bat Protection and Information, Bad Segeberg, Germany; 9 Kumasi Centre for Collaborative Research in Tropical Medicine (KCCR), Kumasi, Ghana; 10 Kwame Nkrumah University of Science and Technology, Kumasi, Ghana; 11 Centre de Cooperation Internationale de Recherche en Agronomie pour le Développement, UPR AGIRs, Montpellier, France; 12 Department of Infectious Diseases, Molecular Virology, Medical Facility, Heidelberg University, Heidelberg, Germany; 13 Friedrich-Loeffler-Institut, Institute for Virus Diagnostics, Greifswald–Insel Riems, Germany; 14 Friedrich-Loeffler-Institut, Institute for Novel and Emerging Infectious Diseases, Greifswald–Insel Riems, Germany; 15 Institute of Medical Virology (Helmut Ruska Haus), Charité Medical School, Berlin, Germany; 16 Bernhard Nocht Institute for Tropical Medicine, Department of Virology, Hamburg, Germany; 17 Unidad de Investigación Médica en Inmunología, Hospital de Pediatría, México DF, Mexico; 18 Chulalongkorn University, Faculty of Medicine, Neuroscience Center for Research and Development, Bangkok, Thailand; 19 Institute of Virology, Free University of Berlin, Department of Veterinary Medicine, Berlin, Germany; 20 Department of Conservation Ecology and Entomology, Stellenbosch University, Stellenbosch, South Africa; 21 Netherlands Center for Infectious Disease Control, Bilthoven, The Netherlands; 22 Centre International de Recherches Médicales de Franceville, Franceville, Gabon; 23 Institut de Recherche pour le Développement, UMR 224 (MIVEGEC), IRD/CNRS/UM1, Montpellier, France; Washington University, United States of America

## Abstract

Hepatitis C virus (HCV) is among the most relevant causes of liver cirrhosis and hepatocellular carcinoma. Research is complicated by a lack of accessible small animal models. The systematic investigation of viruses of small mammals could guide efforts to establish such models, while providing insight into viral evolutionary biology. We have assembled the so-far largest collection of small-mammal samples from around the world, qualified to be screened for bloodborne viruses, including sera and organs from 4,770 rodents (41 species); and sera from 2,939 bats (51 species). Three highly divergent rodent hepacivirus clades were detected in 27 (1.8%) of 1,465 European bank voles (*Myodes glareolus*) and 10 (1.9%) of 518 South African four-striped mice (*Rhabdomys pumilio*). Bats showed anti-HCV immunoblot reactivities but no virus detection, although the genetic relatedness suggested by the serologic results should have enabled RNA detection using the broadly reactive PCR assays developed for this study. 210 horses and 858 cats and dogs were tested, yielding further horse-associated hepaciviruses but none in dogs or cats. The rodent viruses were equidistant to HCV, exceeding by far the diversity of HCV and the canine/equine hepaciviruses taken together. Five full genomes were sequenced, representing all viral lineages. Salient genome features and distance criteria supported classification of all viruses as hepaciviruses. Quantitative RT-PCR, RNA in-situ hybridisation, and histopathology suggested hepatic tropism with liver inflammation resembling hepatitis C. Recombinant serology for two distinct hepacivirus lineages in 97 bank voles identified seroprevalence rates of 8.3 and 12.4%, respectively. Antibodies in bank vole sera neither cross-reacted with HCV, nor the heterologous bank vole hepacivirus. Co-occurrence of RNA and antibodies was found in 3 of 57 PCR-positive bank vole sera (5.3%). Our data enable new hypotheses regarding HCV evolution and encourage efforts to develop rodent surrogate models for HCV.

## Author Summary

The hepatitis C virus (HCV) is one of the most relevant causes of liver disease and cancer in humans. The lack of a small animal models represents an important hurdle on our way to understanding, treating, and preventing hepatitis C. The investigation of small mammals could identify virus infections similar to hepatitis C in animals that can be kept in laboratories, such as rodents, and can also yield insights into the evolution of those ancestral virus lineages out of which HCV developed. Here, we investigated a worldwide sample of 4,770 rodents, 2,939 bats, 210 horses and 858 cats and dogs for HCV-related viruses. New viruses were discovered in European bank voles (*Myodes glareolus*) and South African four-striped mice (*Rhabdomys pumilio*). The disease in bank voles was studied in more detail, suggesting that infection of the liver occurs with similar symptoms to those caused by HCV in humans. These rodents might thus enable the development of new laboratory models of hepatitis C. Moreover, the phylogenetic history of those viruses provides fascinating new ideas regarding the evolution of HCV ancestors.

## Introduction

Hepatitis C virus is one of the leading causes of human morbidity and mortality due to hepatitis, liver cirrhosis, and hepatocellular carcinoma [1], [2], [3]. It has become the main reason for liver transplantation in developed countries and represents an economic burden exceeding 1 billion US$ of direct health costs [4], [5]. New estimates of the burden of disease suggest at least 185 million individuals worldwide to have been seropositive in 2005, with a tendency to increase [6]. Treatment has considerably improved due to the optimization of antiviral regimens and the advent of new antiviral drugs [7], [8], [9]. However, treatment in resource-limited settings is hardly accessible [10]. The most effective instrument to prevent new infections with HCV would be a prophylactic vaccine. Unfortunately, chimpanzees are the only known animal species to adequately reflect human HCV infection [11]. Vaccine development is hampered by the lack of a small animal model accessible at early stages of vaccine development [12], [13]. Mice cannot be infected with HCV [14], but rats and mice engrafted with human hepatoma cells or transgenic for human CD81 and other co-receptor molecules have been proposed [12], [15], [16], [17]. Mouse-adapted HCV has also been generated [16], [18]. Still, these models are highly demanding from a technical point of view and reflect only parts of the pathogenesis and lifecycle of HCV, precluding their wide application [12], [19].

A HCV-related hepacivirus of unknown origin, termed GBV-B, has been used as a surrogate model for HCV infection involving New World monkeys, where it causes hepatitis upon experimental inoculation [20], [21]. The use of a surrogate model based on a related virus indicates a way to study HCV pathogenesis and immunity, even though neither monkeys nor apes are acceptable laboratory models in terms of accessibility and ethics [12], [13], [22]. Non-Primate hepaciviruses related to HCV have also been detected in dogs and horses [23], [24]. While horses cannot be considered as laboratory models, dogs at least have compatible body sizes. However, additional to ethical controversies, infected dogs showed grossly deviating pathology in that they appeared to have higher virus concentrations in respiratory specimens than in the liver [23]. So far there is no evidence of antibodies against the virus in dogs, limiting their utility as a vaccination challenge model [23], [24]. No hepaciviruses have been detected in other animals that could be kept in laboratories with reasonable effort, and under ethically acceptable conditions.

The targeted identification of animal hepaciviruses might help elucidating the obscure origins of HCV and yield more accessible HCV surrogate models. We have recently demonstrated that the systematic investigation of small mammal reservoirs can yield novel viruses that are genetically closely related to human pathogenic viruses, such as the paramyxoviruses mumps and Nipah virus [25]. Biological and ecological considerations direct research interests to animals with properties supportive of virus maintenance. The close social interaction of certain bat species forming large and dense social groups favors virus maintenance [25], [26]. Virus spreading by bats may be facilitated by their migratory lifestyle, but also by human activities such as hunting of bats as bushmeat and human invasion of remote habitats [27], [28], [29]. Several rodent species are also in focus as potential virus reservoirs, as they constitute habitat generalists and follow human civilization, providing opportunities for virus transmission [30], [31]. Even though rodents form smaller social groups than bats, some rodent species have a high population turnover, which should enable efficient maintenance of viruses through the continuous replenishment of susceptible individuals [26], [32]. Among terrestrial mammals, rodents and bats together constitute about two thirds of the 5,487 known mammalian species [33]. Screening of wild mammals with a view on laboratory models should be oriented by criteria such as small body size and the ability to adapt to laboratory conditions, which applies to rodents, but not bats [12], [34]. Here we have investigated 7,709 bats and rodents pertaining to 92 species sampled globally in ten tropical and temperate countries. The investigation was complemented by a comparison of virus diversity in 1,068 horses, cats and dogs.

## Materials and Methods

### Ethics statement

All animals were handled according to national and European legislation, namely the EU council directive 86/609/EEC for the protection of animals. For all individual sampling sites, study protocols including trapping, sampling and testing of animals were approved by the responsible animal ethics committees as detailed below. All efforts were made leave animals unharmed or to minimize suffering of animals. Any surgical procedure was performed under sodium pentobarbital/ketamine anesthesia. Trapping of rodents in Germany was conducted in the framework of hantavirus monitoring activities and was coordinated by the Friedrich-Loeffler-Institut, the Federal Research Institute for Animal Health. Rodent monitoring in the federal states Mecklenburg-Western Pomerania, Thuringia, Baden-Wuerttemberg and North Rhine Westphalia was coordinated by the Julius Kühn Institute (permit numbers LALLF M-V/TSD/7221.3-2.1-030/09, TH 15-107/09, BW 35-9185.82/0261 and NW 20.09.210. Additional animals were provided by forest institutions and pest management institutions in Mecklenburg-Western Pomerania, Thuringia, Brandenburg, Lower Saxony, Baden-Wuerttemberg, Berlin and Budapest which caught and sacrificed the animals during their official duties without necessity of further permits. Rodents and other small mammals trapped by cats were included in the investigations. Rodent sampling in South Africa was licensed by Cape Nature under permit numbers 317/2003 and 360/2003. Rodent sampling in The Netherlands was licensed by the Dutch animal ethic committee (DEC) under permit numbers 200700119, 200800113 and 200800053. Rodent sampling in Thailand was granted by the Agricultural Zoology Research Group, Department of Agriculture, Thailand (permit no. KU./14-182). Rodent sampling in Gabon was licensed by the Ministry of Water and Forest, statement 003/MEF/SG/DGEF/DFC from 2011. Rodent sampling in Mexico was licensed by Secretaría del Medio Ambiente del gobierno de México (SEMARNAT) under permit number SGPA/DGVS/08283/12. Horse, dog and cat samples were collected from regular diagnostic specimens sent to the OIE Reference Laboratory for Equine Influenza and Herpesviruses at the Freie Universitaet Berlin. Sampling and capture of bats as well as sample transfers were done under wildlife permits and ethics clearances: Panama (Research-Permit STRI: STRI2563 (PI VC) - IACUC 100316-1001-18/Research-Permit ANAM: SE/A-68-11/Ethics-Permit: IACUC 100316-1001-18/Export Permits: SEX/A-30-11, SEX/A-55-11, SEX/A-81-10, SEX-A-26-10); Ghana (Research Permit: 2008–2010 (A04957)/Ethics-Permit: CHRPE49/09/Export-Permit: State Agreement between Ghana and Hamburg (BNI)); Australia (Research Permit: S11828 and S11762/Ethics-Permit: TRIM 01/1118(2), TRIM 06/3569, and University of Queensland/Animal Ethics Committee SIB600/05/DEST/Export-Permit: DE201-12); Papua-New Guinea (Ethics-Permit: PNG/NatMus/2002/Export-Permit: Conducted by Papua New Guinea National Museum); Gabon (Ethics-Permit: 00021/MEFEPA/SG/DGEF/DFC); Germany (Ethics-Permit: LANU 314/5327.74.1.6).

### Sampling and RNA purification

For all sampling and exportation of specimens, permission was obtained from the respective authorities (see Acknowledgement for individual permits). Animals were caught with mist nets, live or snap traps, identified by trained field biologists on site or prior to dissection (where applicable), euthanized and dissected in the respective laboratories. Canine, feline and equine samples were routine diagnostic specimens. Between 10–140 µL of blood were extracted using the Qiagen Viral RNA Mini kit (Qiagen, Hilden, Germany). Approximately 30 mg of solid organ tissue were homogenized in a TissueLyser (Qiagen) and purified using the RNeasy Kit (Qiagen).

### Hepacivirus detection and quantification

Six nested PCR assays for amplification of hepacivirus RNA and two assays targeting the *Flaviviridae* sister-genera *Flavivirus* and *Pestivirus* were used to ensure broad detection. Highly sensitive HCV-specific assays targeting the X-tail, NS5B and 5′-untranslated genomic regions were used in addition (see **Supplementary Table S2** for oligonucleotide sequences and reaction conditions). RNA quantification relied on strain-specific real-time RT-PCR assays and photometrically quantified *in vitro* RNA transcripts generated as described previously [35].

### Full genome sequencing

No isolation attempts were made due to the small available specimen quantities and notorious difficulty of hepacivirus isolation. Instead, those rodent specimens with highest RNA concentrations were selected for full genome sequencing. Genome-spanning islets were amplified by PCR using degenerate broadly reactive oligonucleotides (**Supplementary Table S2**). Bridging strain-specific oligonucleotide primers (available upon request) were then designed to perform long range PCR using the Expand High Fidelity kit (Roche) on cDNA templates generated with the SuperScriptIII kit (Invitrogen). Some cDNA templates were enriched using a Phi29-based hexamer-driven amplification using a modified protocol of the Qiagen Whole Transcriptome Amplification kit (Qiagen) as described previously [25]. Amplicons were Sanger sequenced using a primer walking strategy. The 5′-genome ends were determined using the Roche rapid amplification of cDNA ends (RACE) kit (Roche) generating contiguous PCR amplicons encompassing the complete 5′-untranslated region (5′-UTR) and the 5′-terminus of the *core* gene. 454 junior next generation sequencing was used for confirmation of 5′-UTR sequences. For determination of the 3′-genome end, viral RNA was adenylated using a poly-A-polymerase (Clontech, Paris, France) followed by 3′-RACE using the Invitrogen GeneRacer Kit (Invitrogen).

### Phylogeny

Bayesian tree topologies were assessed with MrBayes V3.1 [36] using the WAG amino acid substitution matrix and BEAST V1.7.4 [37] using the GTR model for nucleotide sequences and the FLU model for amino acid sequences. For MrBayes, two million MCMC iterations were sampled every 100 steps, resulting in 20,000 trees. For BEAST, 10,000,000 generations run under a strict clock were sampled every 1,000 steps, resulting in 10,000 trees. Burn-in was generally 25% of tree replicates. A human pegivirus (previously termed GBV-C1; GenBank, U36380) was used as an outgroup. Maximum Likelihood analyses were used to confirm Bayesian tree topologies using the WAG amino acid substitution model and 1,000 bootstrap replicates in PhyML [38]. Trees were visualized in FigTree from the BEAST package and Densitree [39].

### Folding

RNA secondary structures in viral 5′- and 3′-genome ends were inferred manually basing on covariant base pairing and thermodynamic predictions using mfold [40] in an alignment of rodent, primate and canine/equine hepaciviruses generated with MAFFT [41].

### Prediction of signal peptidase cleavage and N-/O-glycosylation sites

Putative cellular signal peptidase (SP) cleavage sites were predicted based on artificial neural networks (NN) and hidden Markov models (HMM) using the SignalP 3.0 Server [42]. N- and O-glycosylation sites were determined using the online tools NetNGlyc 1.0 Server and NetOGlyc Server [43], [44].

### Genome comparison

Putative genes were annotated based on predicted signal peptidase (SP) cleavage sites (where applicable) and sequence homology to HCV, GBV-B and canine/equine hepaciviruses. Alignments were generated using MAFFT [41]. Amino acid percentage identity matrices were calculated using MEGA5 [45] with the pairwise deletion option.

### Statistics

Comparison of mean virus concentrations was done using an ANOVA analysis with Scheffé post-hoc tests in the SPSS V20 software package (IBM, Ehningen, Germany). Cross-tables were done using EpiInfo7 (www.cdc.gov/epiinfo).

### Serology

#### HCV Western blot

Western blot (WB) analysis was performed with commercially available HCV strips (recomBlot HCV IgG 2.0 and recomLine HCV IgG, Microgen, Neuried, Germany). Bat and rodent sera were diluted 1∶100 for screening. Horseradish peroxidase-labelled goat anti-bat immunoglobulin (Ig) conjugate (Bethyl, Montgomery, AL, USA) or goat-anti mouse Ig (Dianova, Hamburg, Germany) were used as secondary antibodies (dilution, 1∶500). For rodent WB, a tertiary horseradish peroxidase-labelled donkey-anti goat Ig (Santa Cruz Biotechnology, Santa Cruz, CA, USA) was used for signal amplification. Blots were evaluated following the manufacturer's instructions.

#### HCV immunofluorescence assay

An indirect immunofluorescence assay (IFA) was done using HCV-infected HuH7-cells (strain JC1) or replicon JFH1-transfected cells. Cells were fixed with paraformaldehyde (4%), permeabilized with 0.5% Triton X-100 in 1×PBS for 5 minutes and processed as described previously [46]. Bat sera were diluted 1∶50. Reactions were detected with goat-anti-bat Ig (Bethyl, 1∶1000) and cyanine 2 (Cy2)–labelled donkey-anti-goat Ig (Dianova, 1∶100). For control reactions, a polyclonal rabbit NS3-Ig raised against the NS3 helicase domain of JFH1 (1∶400) and an Alexa 568-conjugated goat-anti-rabbit Ig (Invitrogen, 1∶1000) were used.

#### Rodent hepacivirus immunofluorescence assay

VeroFM cells were transfected in suspension using FuGENE HD (Promega, Mannheim, Germany) with 0.75 µg plasmid expressing the complete His-tagged NS3 proteins of the rodent hepaciviruses RMU10-3382 (rNS3RMU10-3382) and NLR-AP70 (rNS3AP70) and fixed 24 hours later with acetone/methanol (80%/20%). *Myodes glareolus* sera were tested at screening dilutions of 1∶10 and 1∶40. For secondary detection, a goat-anti-mouse Ig (Dianova, 1∶2000) and a donkey-anti-goat cyanine 3-labelled Ig (Dianova, 1∶200) were applied. Recombinant rNS3RMU10-3382 protein including a cleavable Thioredoxin/His_6_ tag was expressed in bacteria and purified under non-denaturing conditions following a standard protocol [47]. The untagged purified protein was used to produce specific rabbit polyclonal antisera at Thermo Scientific Pierce custom antibody service. Rabbit antiserum against rNS3RMU10-3382 (1∶2000) was used in parallel to an rNS3RMU10-3382-reactive rodent serum (1∶50) for a co-localization study by confocal laser scanning microscopy. Here, secondary detection was performed using a cyanine 2-labelled goat-anti rabbit Ig (Dianova, 1∶200) and a cyanin 3-conjugated goat-anti-mouse Ig (Dianova, 1∶200).

### In-situ hybridization

RNAScope RNA probes targeting a 978 nucleotide NS3 gene fragment of the *M. glareolus* clade 1 hepacivirus detected in specimen RMU10-3379 were custom designed by Advanced Cell Diagnostics (Hayward, CA, USA). RMU10-3379 was selected due to best tissue quality and high virus concentration. In-situ hybridization was performed as described by the manufacturer.

### Accession numbers

All virus sequences reported in this study were submitted to GenBank under accession numbers KC411776-KC411814.

## Results

**Figure 1:**
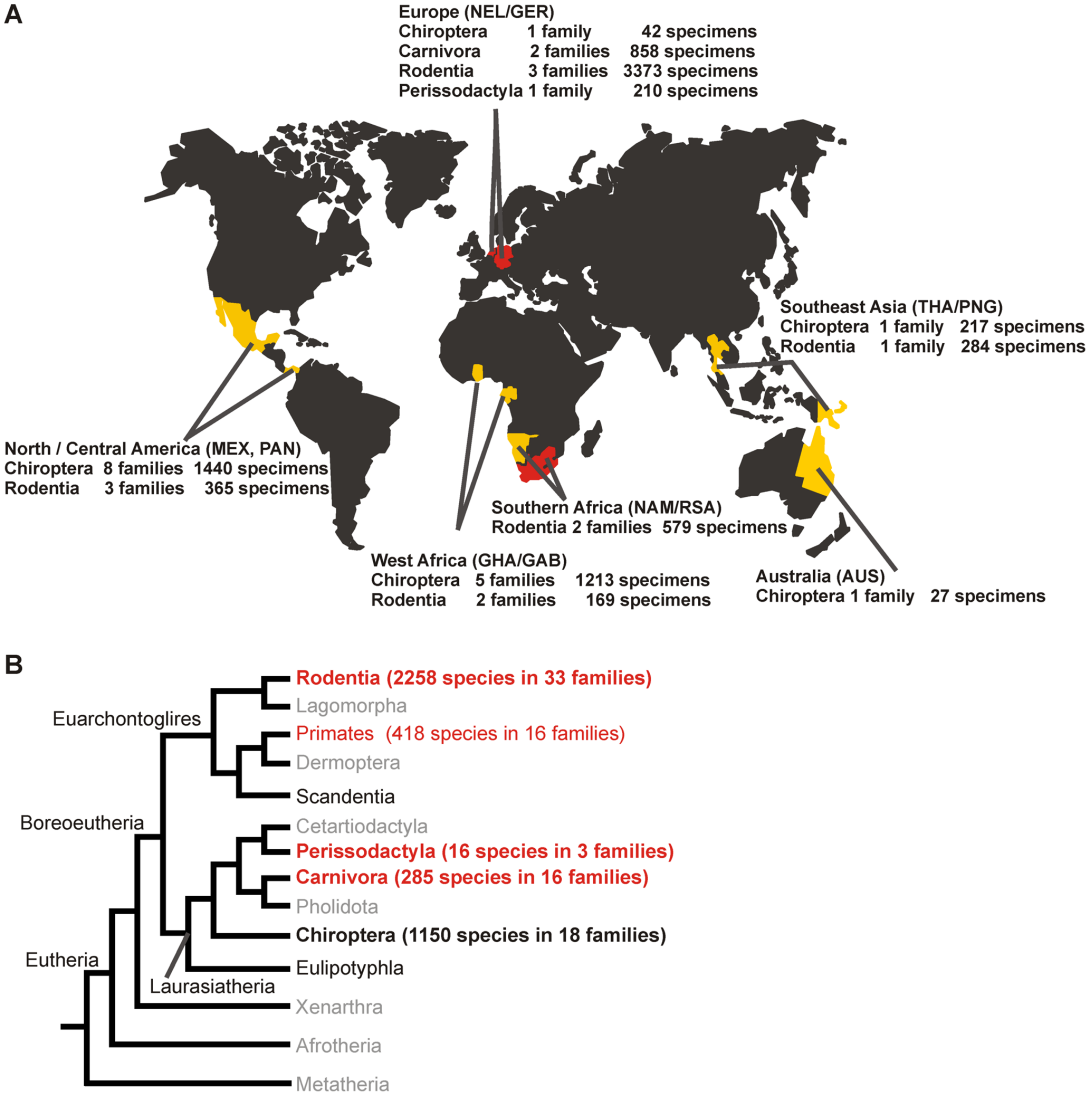
Sampling sites, years, species and families. A. Sampling sites yielding novel hepaciviruses are indicated in red, others in yellow. B. Placentalia (Eutheria) evolutionary lineages according to [76]. Major mammalian clades are identified at basal nodes of the Placentalia phylogeny: Afrotheria (e.g., elephants), Xenarthra, (e.g., anteaters) and Boreoeutheria, divided into the two superorders Euarchontoglires, (e.g., primates, rodents) and Laurasiatheria (e.g., dogs, bats). Sampled mammalian orders are shown in boldface type. Orders containing novel hepaciviruses identified in this study are shown in red and boldface. Orders with known hepaciviruses (perissodactyla, primates, carnivora) are given in red. Numbers of extant families and species per order adapted from [33] are indicated.

Specimens from 8,777 individual animals from the orders Chiroptera, Rodentia, Carnivora and Perissodactyla were included in this study. The geographical origins of samples are summarized in **Figure 1AB**. The sample contained sera and liver tissue from 4,770 rodents (Rodentia, 41 species), sera from 2,939 bats (Chiroptera, 51 species), sera from 210 horses (Perissodactyla) and sera from 167 dogs (Carnivora). Due to the reported respiratory tropism of canine hepaciviruses, snout swabs were additionally obtained from 239 dogs and 452 cats. The detailed composition of the sample is listed in **Supplementary Table 1**.

### Pre-screening by serology

**Figure 2:**
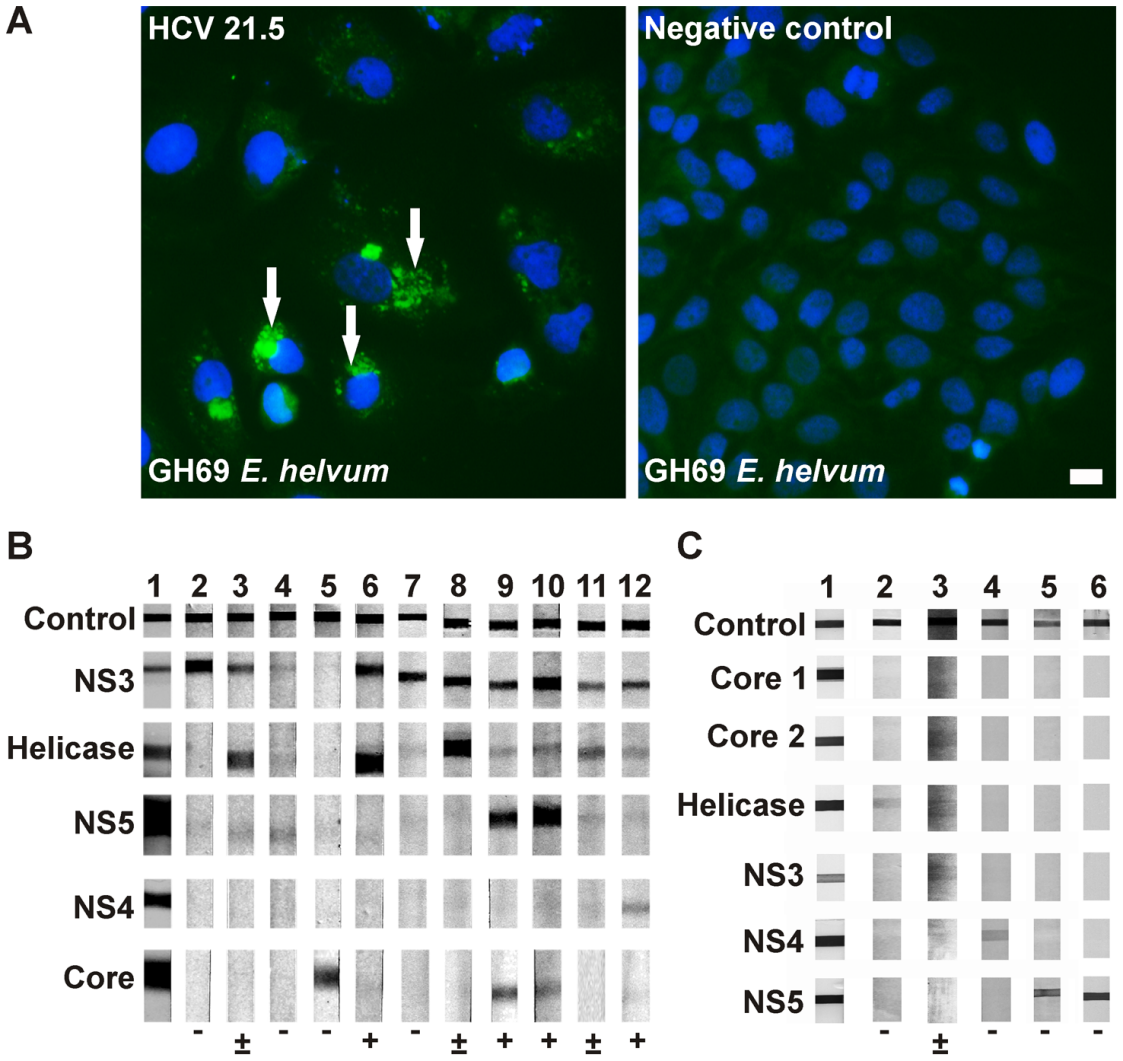
Serological reactivity of bat and rodent sera with HCV antigens. A. Indirect immunofluorescence assay using bat serum. Typical reactivity of a positive *E. helvum* serum from Ghana (GH69) diluted 1∶50 in sample buffer with HuH7 cells infected with HCV strain JHF1 is shown on the left. Arrows point at specific staining of cytoplasmatic antigen. On the right, lack of reactivity of GH69 with uninfected HuH7 cells is shown. IFA was done as described in the methods section. Cell nuclei were stained with DAPI. Scale bar represents 100 µm. B. HCV western blot reactivity patterns with bat sera. Representative reaction patterns of 11 bat sera with the HCV recomblot assay are shown. Sample 1, human positive control serum. Samples 2 to 12 correspond to the following bat species: 2–7, *Eidolon helvum*; 8–12, *Rousettus aegyptiacus*. C. HCV western blot reactivity patterns with rodent sera. Representative reaction patterns of 5 rodent sera with the HCV recomline are shown. Sample 1, human positive control serum. Samples 2 to 6 correspond to the following rodent species: 2, *Rattus norvegicus*; 3, *Apodemus sylvaticus*; 4, *Myocastor coypus*; 5, *Rattus norvegicus*; 6, *Myodes glareolus*. Blot antigens are indicated at the left of each row. Below each line in B and C, the result of a tentative evaluation is given following the manufacturer's criteria defined for human sera, as described below Table 1.

Enough serum for serologic testing was available from 180 bats (72 *Rousettus aegyptiacus* and 108 *Eidolon helvum*) and 95 rodents (33 *Myodes glareolus*, 30 *Apodemus sylvaticus*, 30 *Rattus norvegicus*, 2 *Myocastor coypus*). In initial tests using immunofluorescence slides containing full recombinant HCV, 13 bats (7.2%, 9 *R. aegyptiacus* and 4 *E. helvum*) showed reactivity patterns suggestive of antibodies cross-reacting with HCV. **Figure 2A** exemplifies typical IFA reaction patterns observed. For confirmation, recombinant HCV western blot (WB) assays certified for diagnostic application in humans were adapted for use with bat and rodent sera. For 95 of the 180 bat sera, enough serum volume for WB testing was available. This included three of the 13 IFA-positive sera. As shown in **Table 1**, between seven (Core) to 28 (NS3/Helicase) sera were clearly reactive with different WB antigens. 10 sera (10.6%) were to be interpreted as antibody-positive upon criteria for the interpretation of western blot results applicable in human diagnostics. **Figure 2B** provides examples of typical reaction patterns. The three IFA-positive sera were also positive in WB. For rodents, **Table 1** shows that two (Helicase and NS4) to six (NS5B) sera reacted with individual antigens. Another 45 sera showed borderline reactivities comparable to the intensity of the WB cut-off control (examples of reactivities in **Figure 2C**). No rodent sera fulfilled the criteria for positive interpretation applicable in human diagnostics.

**Table 1:** Western blot reactivity patterns.

	Interpretation*				Reactivities by individual antigens														
Western Blot					NS3			Helicase			NS5			NS4			Core		
Bat species	N	+	±	−	+	±	−	+	±	−	+	±	−	+	±	−	+	±	−
*Eidolon helvum*	89	7	18	64	22	39	28	26	36	27	9	35	45	6	22	61	5	20	64
*Rousettus aegyptiacus*	5	3	2	0	5	-	1	2	3	1	2	1	3	1	2	3	2	1	3
**Total (percent)**	94	10 (10.6)	20 (21.3)	64 (68.1)	27	39	29	28	39	28	11	36	48	7	24	64	7	21	67
**Rodent species**																			
*Myodes glareolus*	33	0	1	33	-	-	-	1	5	-	2	7	-	-	2	-	-	-	-
*Apodemus sylvaticus*	30	0	1	29	-	1	-	-	2	-	1	7	-	-	4	-	-	1	-
*Rattus norvegicus*	30	0	0	30	-	-	-	1	2	-	3	8	-	-	6	-	-	-	-
*Myocastor coypus*	2	0	0	2	-	-	-	-	-	-	-	-	-	2	-	-	-	-	-
**Total (percent)**	95	0	2 (2.1)	94 (97.9)	0	1	0	2	9	0	6	22	0	2	12	0	0	1	0

* Blot analysis criteria for human diagnostics provided by the manufacturer (Microgen, Neuried, Germany): A positive band was assigned the following point values: NS3, 3; Helicase, 3; NS5-12, 2; NS4, 4; Core, 8. Only weakly positive bands (visible, but weaker than the core antigen of the weak positive HCV control) were considered negative with the exception of the core antigen, which was then assigned 5 points. Blots were considered positive if the sum of points was equal to or greater than 10, borderline if the sum was between 6 and 9 points, and negative if the sum was equal to or below 5 points. Eight bat sera showing strongest Western Blot reactivity were end-point diluted to evaluate specificity of the reaction. Bands remained visible up to a dilution of 1∶400. Some analyses were done using a follow-up version of the Microgen assay (recomline) with two separate core antigens and different interpretation criteria. Blots were then considered borderline if any two antigens showed higher intensity than a cut-off control, the helicase alone, the helicase and any NS antigen or any core epitope. Blots were considered positive in this assay version if both core antigens were positive, one core plus any other antigen or if three antigens showed higher intensities than the cut-off control. Reactivity of the secondary goat anti-mouse and tertiary donkey anti-goat antibodies used for rodent testing were controlled by using mouse anti-core and anti-NS3 monoclonal antibodies for primary reaction with blot antigens.

### Hepacivirus detection

For the molecular analysis of bats, 2,939 sera from Gabon, Ghana, Papua-New Guinea, Australia, Thailand, Panama and Germany were tested for *Hepacivirus* RNA using several broadly reactive and highly sensitive RT-PCR assays, as detailed in **Supplementary Table 2**. Despite the apparent relatedness of putative bat hepaciviruses with HCV suggested by the serologic analyses, no hepacivirus RNA was detected in any of the specimens, whereas several PCR fragments from the NS3 gene were obtained which upon sequencing were identified as pegiviruses related to GBV-D [48].

Tested rodent specimens originated from Thailand, Gabon, South Africa, Germany, the Netherlands and Mexico (**Supplementary Table 1**). HCV-related sequences from the NS3 gene were detected in 37 out of 4,770 specimens (0.8%). Ten of these findings were from South African four-striped grass mice (*Rhabdomys pumilio*; 10 of 518 individual animals, 1.9%). For these and all other positive specimens, a 978 nucleotide NS3 fragment was generated using additional primer pairs (**Supplementary Table 2**). The derived sequences pertained to one clade, and were different from each other by 21.1% on nucleotide, or 3.4% on translated amino acid level. Twenty-seven (1.8%) of 1,465 individual bank voles (*Myodes glareolus*) from Germany and The Netherlands yielded HCV-related NS3 sequences. The derived sequences fell into two separate clades. Clade 1 contained 23 sequences different from each other by up to 15.8% of nucleotides and 2.8% of translated amino acids. Clade 2 contained four sequences different by 1.6% nucleotides and 0.6% translated amino acids.

**Figure 3:**
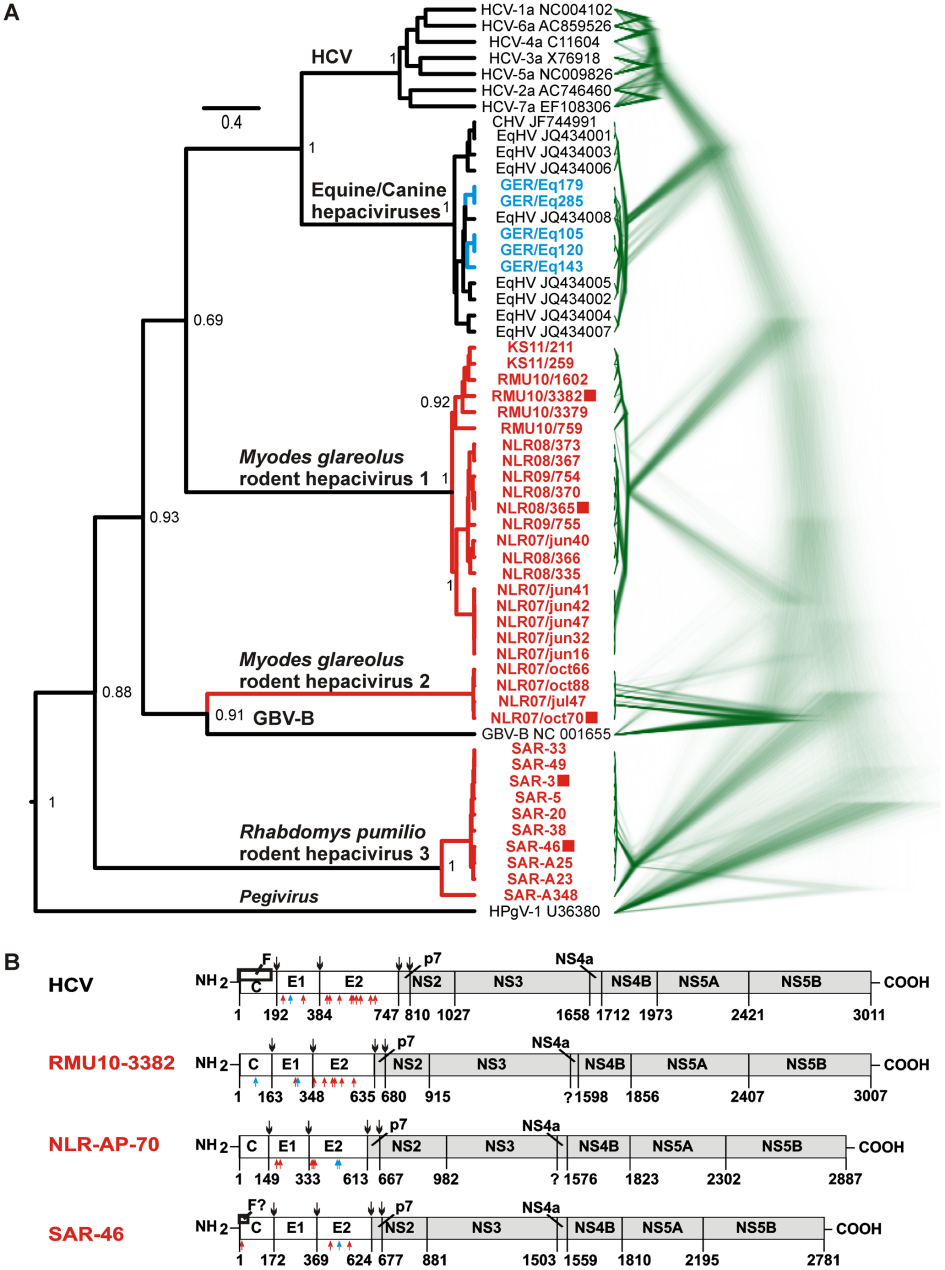
Genomic characterization of the novel rodent hepaciviruses. A. Partial NS3 gene phylogeny. The analysis comprised a 978 nucleotide fragment of the HCV NS3 gene corresponding to positions 3,912–4,889 in HCV 1a H77 (GenBank, NC_004102). GenBank accession numbers of reference hepaciviruses are indicated to the right of taxon names. Tree topology was inferred using BEAST with a GTR nucleotide substitution model as described in the methods section. Rodent hepaciviruses from this study are shown in red and boldface, equine hepaciviruses from this study are shown in blue and boldface. Red squares indicate those viruses whose near full-length genomes were generated. Statistical support of grouping is shown as posterior probabilities at deep nodes. Scale bar corresponds to genetic distance. To the right, 5,000 tree replicates of the same analysis are rendered using Densitree (initial 5,000 trees discarded as burn-in). Green line color indicates low probability of all trees, line thickness corresponds to concordant topologies across tree replicates. B. Genome organization of the novel rodent hepaciviruses. Genes were annotated as described in the methods section. Black arrows on the top indicate predicted signal peptidase cleavage sites. Red arrows below indicate N-, blue arrows O-glycosylation sites. Putative gene starts and ends are numbered below polyprotein plots. HCV 1a strain H77 is depicted on top as a reference. RMU10-3382 (KC411777) also represents the highly similar virus NLR-365 (KC411796) in *M. glareolus* hepacivirus clade 1. SAR-46 (KC411807) also represents SAR-3 (KC411806), both from the *R. pumilio* hepacivirus clade. GenBank accession number of NLR-AP70 representing *M. glareolus* hepacivirus clade 2 is KC411784. The structural genome region included *Core* (C), *Envelope* 1 and 2 (E1 and E2) and p7 genes. The boxes in the HCV and SAR-46 *Core* gene indicate a putative F protein open reading frame. The putative *Core* gene at the N′-terminus of the rodent hepacivirus polyproteins included a high number of strongly basic lysine and arginine residues (*M. glareolus* clade 1, 29 of 163 residues (17.8%); *M. glareolus* clade 2, 23 of 149 (15.4%); *R. pumilio* clade, 28 of 172 (16.3%); compared to 31 of 191 (16.2%) in HCV-1a and 11 of the N′-terminal 200 residues (5.5%) in the pegivirus GBV-C1 not encoding a Core protein. Non-structural genes included an NS3 *protease*/*helicase* gene, the phosphoprotein NS5a and the NS5b gene encoding the *RNA-dependent RNA polymerase*. Within the NS4 gene, only the NS4b portion could be clearly identified for all viruses. An NS4a homologue could only be detected in virus SAR-46. IRES types and structural elements in the 3′-genome ends are depicted adjacent to the polyprotein plots for viruses NLR-3382 and SAR-46. Of NLR-AP70, only the 5′-end could be partially determined. This sequence was almost identical to RMU10-3382/NLR-365. The 3′-terminus of NLR-AP70 could not be determined.

A Bayesian phylogeny of the partial NS3 gene shown in **Figure 3A** suggested that the *M. glareolus* hepacivirus clade 1 was monophyletic with HCV and the canine/equine hepaciviruses. *M. glareolus* hepacivirus clade 2 was most closely related to GBV-B while the *R. pumilio*-associated clade formed a sister taxon to all other hepaciviruses. An analysis of all replicate trees indicated that the deep phylogenetic nodes were not resolved (**Figure 3A**). The monophyly of HCV and the *M. glareolus* clade 1 hepaciviruses was maintained in 68.6% of tree replicates (3,430 of 5,000). In another 28.9% of trees (1,446/5,000), the two *M. glareolus* hepacivirus clades clustered together. Monophyly of all three rodent hepacivirus clades and GBV-B was indicated in only 15 of 5,000 tree replicates (0.3%).

### Full genome characterization

The near full genomes of five representative hepaciviruses from all rodent clades were determined, including two viruses from *R. pumilio*, two from *M. glareolus* clade 1 and one from *M. glareolus* clade 2 (identified by red squares in **Figure 3A**). The polyprotein genes were of different sizes including 2,781; 2,887; and 3,007 amino acid residues, respectively, compared to 3,008–3,033 in HCV. All genomes shared the typical hepacivirus polyprotein organization, encoding putative proteins in the sequence C-E1-E2-p7-NS2-NS3-NS4A/4B-NS5A-NS5B (**Figure 3B**). The putative structural C, E1, E2 and p7 proteins were predicted by signal peptidase cleavage site analysis (**Supplementary Table S3**) to be comparable in their sizes to that of known hepacivirus proteins. All rodent viruses had considerably fewer predicted glycosylation sites in their structural proteins, in particular their putative E2 proteins, as opposed to HCV. A detailed genome analysis is provided in **Figure 3B**. The 5′-terminus of the core gene of the *R. pumilio* hepacivirus clade contained a putative adenosine-rich slippery sequence at codons 10–14 (AAAAAAAACAAAAA, **Supplementary Figure 3B**). In HCV, a very similar sequence (AAAAAAAAAACAAA), located at nearly the same positions (codons 8–12) of the *core* gene induces production of a protein termed F *in vitro* due to ribosomal frameshift event [49]. Depending on the HCV genotype, the size of the F protein ranges from 126 to 162 amino acid residues which vary considerably in sequence composition [50]. The size of a putative F protein in SAR46 would be 65 amino acid residues and no homology to the HCV F proteins was observed.

The total amino acid diversity of all homologous genes within the polyproteins of the three rodent hepacivirus clades was larger than that of all HCV genotypes (**Supplementary Table S4**). Similar to HCV, the most variable genomic regions in rodent hepaciviruses were located in the *Envelope* E2 gene differing in up to 84.4% of encoded amino acids between the rodent virus clades; the NS2 gene differing in up to 79.8%; and the NS5A gene differing by up to 84.6%.

**Figure 4:**
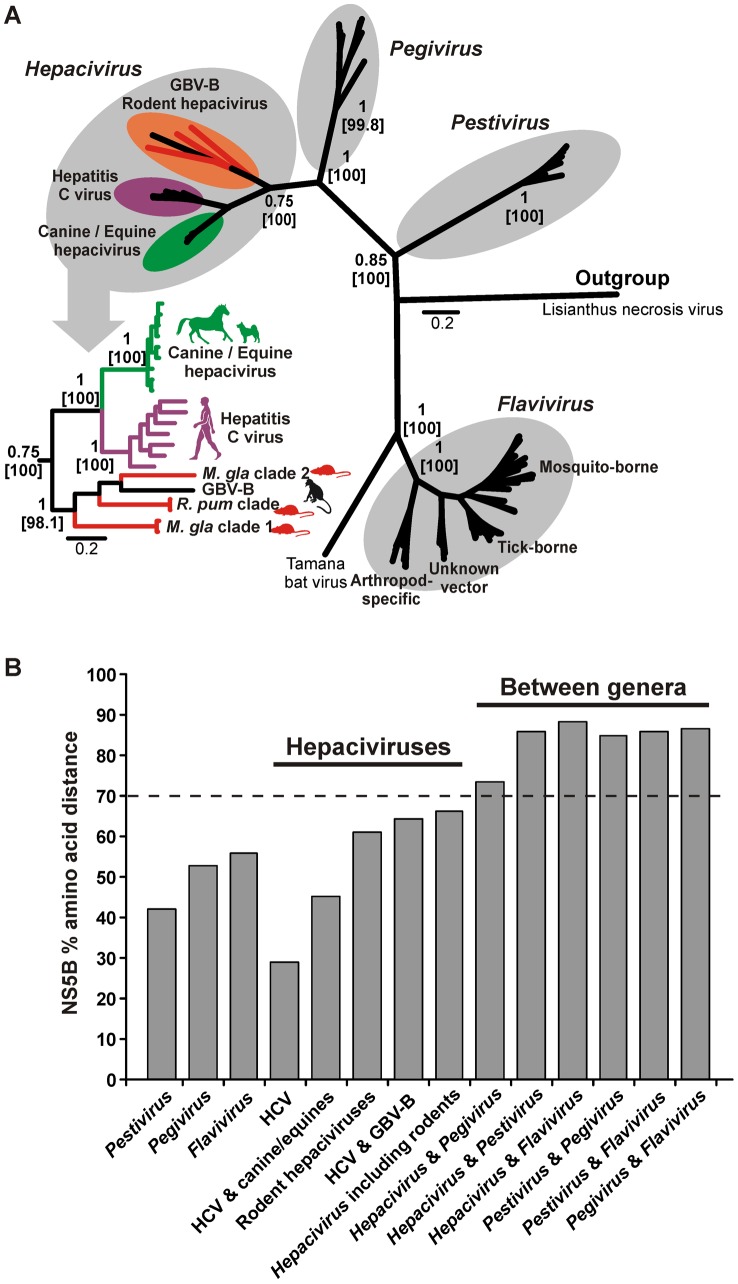
Comparison of the novel rodent hepaciviruses with other ***Flaviviridae*** **.** A. Bayesian phylogeny of the Flaviviridae NS5B gene. The analysis was done in MrBayes and included representatives of all *Flaviviridae* genera and those five novel rodent viruses whose full polyprotein could be determined. The WAG amino acid substitution model was used. Statistical support of grouping from Bayesian posterior probabilities and 1,000 parallel Maximum Likelihood bootstrap replicates is indicated at deep node points. Scale bar corresponds to genetic distance. A tombusvirus (Lisianthus necrosis virus, GenBank accession number NC_007983) was chosen as an outgroup. B. Amino acid distance of the complete NS5B gene within Flaviviridae clades. Maximum amino acid sequence distance was calculated with MEGA5 using the pairwise deletion option and all *Flaviviridae* members contained in panel A. The dotted line indicates 70% distance for clarity of graphical presentation only.

The high degree of sequence homology of the *RNA-dependent RNA polymerase* (*RdRp*) genes between all members of the family *Flaviviridae* enabled a more comprehensive comparison of the novel viruses. In a Bayesian phylogeny of these genes across the flavivirus family, the rodent viruses formed a monophyletic sister-clade to HCV (**Figure 4A**). Topological robustness was assessed by the fixation, in parallel Bayesian phylogenies, of two alternative topological hypotheses, the first involving monophyly of HCV with the canine/equine viruses and *M. glareolus* clade 1, and the second assuming a separation of HCV and the canine/equine viruses from all rodent viruses and GBV-B. A Bayes factor test comparing the total model likelihood traces of these analyses indicated borderline-significant preference of the second hypothesis over the first (Log10 Bayes factor = 2.94). **Figure 4B** provides a comparison of *RdRp-*based amino acid distances within and between *Flaviviridae* genera.

**Figure 5:**
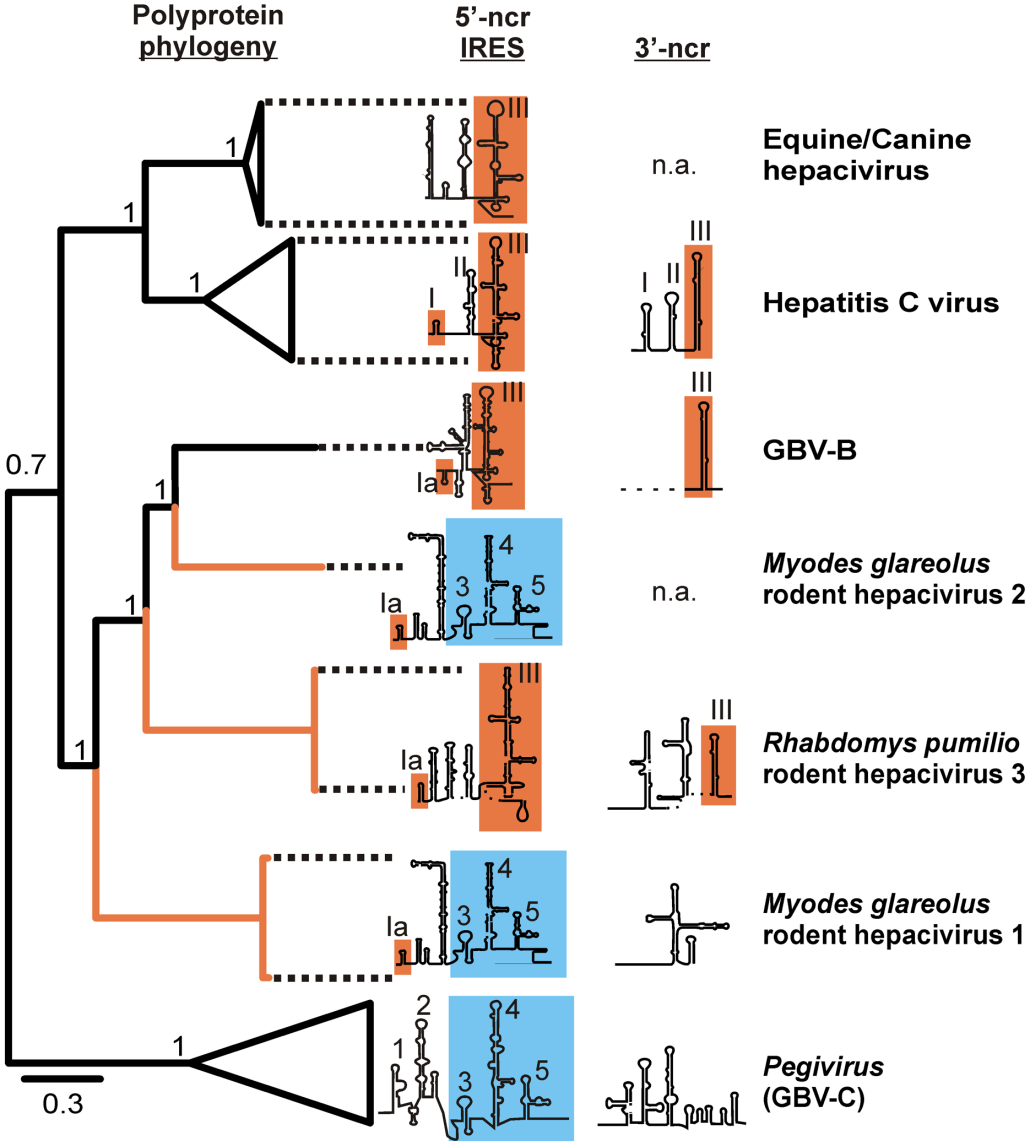
Complete polyprotein gene phylogeny and comparison of the genome termini between the novel rodent and prototype hepaciviruses. For the Bayesian phylogeny shown to the left, the WAG amino acid substitution model was used in MrBayes as indicated in the methods section. Statistical support of grouping from Bayesian posterior probabilities is indicated at node points. Scale bar corresponds to genetic distance. The Pestivirus BVDV (NC_001461) was chosen as an outgroup and truncated for graphical reasons. Branches leading to the novel hepaciviruses from this study are in orange. GenBank accession numbers of analyzed hepaciviruses correspond to those indicated in **Figure 3A**. The 5′- and 3′-genome termini were re-drawn from published foldings for equine hepaciviruses [24], HCV [63], [77] and GBV-B [78] and *de novo* for this study for the 5′- and 3′-ends of GBV-C1 and the 3′-end of GBV-B (see **Supplementary Figure 4**). Despite earlier attempts to fold the 3′-ncr of GBV-B [21] only the 3′-terminal stem-loop structure of GBV-B could be reliably folded due to the single sequence available. The folding of the 3′-end of RMU10-3382 remained tentative for the same reason. No sequence information was available for the 3′-ends of the canine/equine hepacivirus clade and *M. glareolus* hepacivirus clade 2 (indicated as “n.a.”). Typical pegivirus domains are highlighted in blue and ordered by arabian numbers. Typical hepacivirus domains are highlighted in orange and numbered by roman numbers.

In a Bayesian phylogeny of the full polyprotein, the rodent hepaciviruses and GBV-B were monophyletic, forming a sister clade to the canine/equine hepaciviruses and HCV (**Figure 5**). The rodent-associated clade had very long intermediary branches and originated close to the root of all viruses. The full genome tree had a better phylogenetic resolution compared to the partial NS3 phylogeny, but still contained topological uncertainties in some deep nodes leading to rodent-associated taxa (**Supplementary Figure S1**).

The genome ends of representatives of all three rodent viruses were determined, including virus RMU10-3382 belonging to *M. glareolus* clade 1, NLR-AP-70 belonging to *M. glareolus* clade 2, and virus SAR-46 belonging to the *R. pumilio* hepacivirus clade. **Figure 5** and **Supplementary Figure S2A** show that the 5′-genome terminus of RMU10-3382 contained structural elements typical of both pegi- and HCV-like internal ribosomal entry sites (IRESs). Predicted structural similarities with the HCV-like IRES included the first stem-loop element (termed Ia and highlighted in orange in **Figure 5**) and one of two sites involved in miRNA122 binding [51], while most of the remaining stem-loop elements (termed 3, 4 and 5 and highlighted in blue in **Figure 5**) were more closely related to a pegivirus-like IRES. The 5′-end of AP-70 was identical in structure to RMU10-3382 and contained only a few nucleotide exchanges. SAR-46 contained the typical HCV-like IRES structures including the characteristic stem-loop III (**Figure 5** and **Supplementary Figure S2B**). The observed structural similarity between the first stem-loop of all rodent viruses described here and the prototype hepaciviruses HCV and GBV-B consisted of a hairpin with a six-nucleotide stem and four-five nucleotide loop. The equine/canine hepaciviruses contained a similar structural element located as their second predicted IRES domain, instead of the most 5′-position this domain occupied in all other hepaciviruses. The RMU10-3382 and NLR-365 translation initiation sites contained a cytosine immediately following the putative start codon at position +4, which is suboptimal in the original Kozak sequence context (ACCATGG) but should not block initiation [52]. The 3′-ends of RMU10-3382 and SAR-46 contained three highly ordered stem-loop elements. In RMU10-3382, these RNA elements did not resemble any known 3′-noncoding sequence RNA structure. In SAR-46, the 3′-terminal stem-loop structure, but not the preceding structures, resembled that of the HCV X-tail (**Figure 5** and **Supplementary Figure S3**). A similar 3′-terminal structure could be predicted for GBV-B, but not for the genetically related pegiviruses (**Figure 5** and **Supplementary Figure S4**). The 3′-end of NLR-AP-70 could not be determined. Contrary to HCV and GBV-B, no poly-uracil stretch was observed in the rodent hepaciviruses.

### Natural history of hepacivirus infection in bank voles

**Figure 6:**
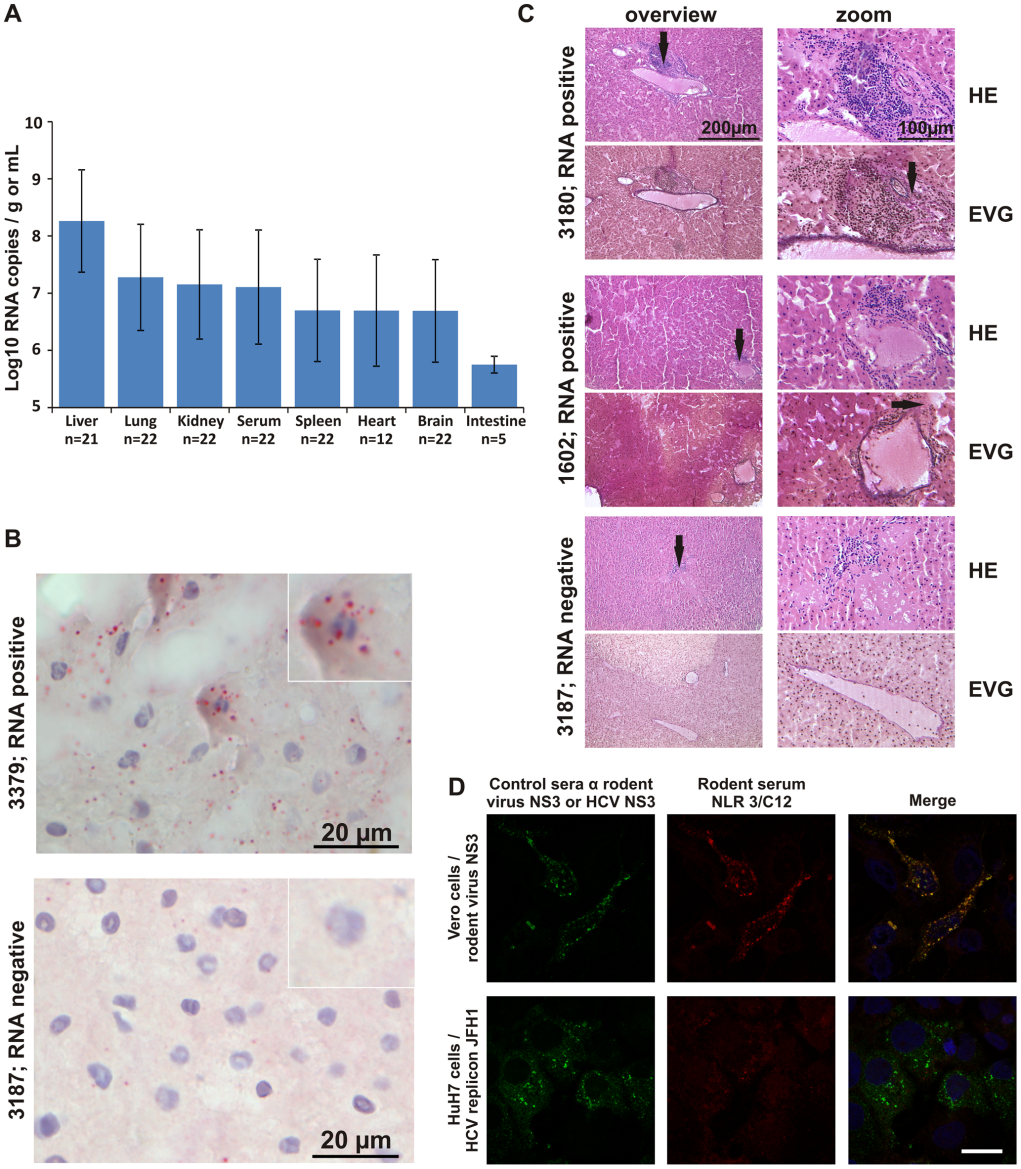
Presentation of hepacivirus infection in bank voles. A. Hepacivirus RNA concentrations in Myodes glareolus. Bars represent means and standard deviation of hepacivirus clade 1- and 2-positive organs and serum. The number of biological replicates is indicated below bars. B. In situ hybridization of rodent hepacivirus clade 1 RNA in M. glareolus. Viral RNA was stained in liver tissue of *M .glareolus* specimen RMU10-3379 (viral RNA concentration, 9.4×10^8^ copies per gram, GenBank accession no. KC411778). The RNA-negative specimen RMU10-3187 from the same species was processed identically and is shown below as a control. Positive staining is visible as distinct red granules in the cytoplasm of hepatocytes. Magnification was 100×, the inserts shows details of single hepatocytes in 10× higher magnification. Scale bars are shown to the lower right. C. Histopathology of M. glareolus liver specimens. Liver sections were stained by Hematoxylin and Eosin (H&E) and Epson van Giesson (EvG) stains. In H&E stains, black arrows point to inflammatory lymphocytic infiltrate. In EvG stains, black arrows highlight potential signs of fibrosis. Specimen 3180 shows intermediate portal inflammatory lymphocytic activity with potential low-grade fibrosis in a case with high hepacivirus RNA concentrations (1.5×10^8^ copies/gram). Specimen 1602 shows low-grade portal inflammatory activity and low-grade fibrosis in a case with high hepacivirus RNA concentrations (3.4×10^8^ copies/gram). Specimen 3187 shows no significantly increased inflammatory activity and no signs of fibrosis in a case with no detectable hepacivirus RNA. Due to highest tissue quality, a terminal hepatic venule instead of a portal triad is shown. D. Recognition of rodent hepacivirus clade 1 antigens by M. glareolus serum. VeroFM cells expressing complete NS3 from *M. glareolus* hepacivirus RMU10-3382 (GenBank, KC411777) were incubated with 1∶2000-diluted rabbit-anti-NS3 3382 antiserum (control) or 1∶50-diluted rodent serum (picture shows exemplary results for animal NLR 3/C12), followed by goat-anti-rabbit-Cy2 (green) and goat-anti-mouse-Cy3 (red) secondary immunoglobulins. For co-localization analysis of fluorescence signals, the 6th of 12 1-µM Z-stags is shown for every channel. Cross-reactivity with HCV antigens was analyzed by incubation of HuH7 cells, transfected with HCV replicon JFH1, with a rabbit-anti-human-HCV-NS3-49 serum, diluted 1∶400 (control) or rodent serum NLR 3/C12 diluted 1∶50, followed by goat-anti-rabbit-Cy2 (green) and goat-anti-mouse-Cy3 (red) secondary antibodies. Counterstaining was performed using DAPI. Bar, 25 µm.

Strain-specific real-time RT-PCR assays were used to determine viral RNA concentrations in tissues of 22 bank voles infected with clade 1 and 2 hepaciviruses. Mean RNA concentrations were highest in liver tissue (1.8×10^8^ copies/gram; range, 1.5×10^6^–4.4×10^9^). These concentrations were significantly higher than those in other organs or serum (ANOVA, F = 7.592, p<0.0001; **Figure 6A** and **Supplementary Figure S5**). **Figure 6B** shows *M. glareolus* clade 1 hepacivirus RNA stained by in-situ hybridization (ISH) in liver tissue. Foci of viral RNA were located in the cytoplasm of *M. glareolus* hepatocytes, while no staining was observed in RT-PCR-negative *M. glareolus* liver specimens (**Supplementary Figure S6** shows additional ISH details). Spleen, kidney, heart and lung tissues yielded no evidence of virus infection by ISH. Histopathological examination of eight RNA-positive and two RNA-negative animals revealed low-grade focal lymphocytic invasion compatible with liver inflammation, such as shown in **Figure 6C** for two exemplary RNA-positive animals.

Serological investigations in wild rodents were complicated by the fact that the vast majority of animals from virus-positive species were not live-trapped, therefore yielding no blood samples. Only post mortem peritoneal lavage fluids were collected from carcasses, but these were not qualified for serology. However, a subset of 97 live-trapped *M. glareolus* with appropriate blood samples were available. These were analyzed for antibodies against the *Myodes* hepacivirus clades 1 and 2 in an IFA using cells expressing the NS3 antigens of these viruses. Antibodies against the *Myodes* hepacivirus clade 1 NS3 antigen were found in eight animals (8.3%) at a median end-point titer of 1∶200 (range, 1∶100–1∶1600). Antibodies against the *Myodes* hepacivirus clade 2 NS3 antigen were detected in 12 animals (12.4%) at a median end-point titer of 1∶600 (range, 1∶100–1∶12800). The difference in antibody detection rates against clades 1 and 2 was not statistically significant (X2 = 0.5, p = 0.5). *Myodes* hepacivirus clade 1 antigen specificity was proven by counterstaining with a high-titered rabbit serum raised against the same recombinant NS3 antigen down to dilutions of >1∶20,000. *Myodes* hepacivirus clade 2 antigen did not cross-react with this rabbit control serum even at high concentrations of 1∶100, compatible with low NS3 amino acid sequence identity between the NS3 proteins of the two *Myodes* hepacivirus clades (42.4%, **Supplementary Table S4**). Neither hepacivirus clade 1, nor clade 2 antibody-positive sera cross-reacted with HCV by immunofluorescence and by immunoblot, indicating specific immune reactions against the viruses studied (exemplary results in **Figure 6D**). This was compatible with low NS3 amino acid sequence identities between both *Myodes* hepacivirus clades and HCV, ranging from 37.9–42.2% (**Supplementary Table S4**). Only one of the eight sera positive against *M. glareolus* clade 1 hepaciviruses also contained antibodies against *M. glareolus* clade 2 hepaciviruses (titers against clade 1 and clade 2 hepaciviruses were 1∶200 and 1∶3200, respectively).

Additional highly sensitive real-time RT-PCR assays were designed specifically for the *M. glareolus* clade 1 and 2 hepaciviruses and used to analyze the association of viral RNA and antibody status in the 97 *M. glareolus* sera. No hepacivirus RNA was detected in any of the IFA-positive sera, neither with the broadly reactive screening assays, nor with the additional real-time RT-PCR assay. Therefore, another 239 RNA eluates still containing sufficient volumes to permit screening for *M. glareolus* clade 1 and 2 hepaciviruses were re-tested with the strain-specific real time RT-PCR assays. Another 57 specimens positive for clade 1 hepaciviruses (23.9%), but no additional clade 2 hepaciviruses were detected. Sera from these PCR-positive animals were obtained and tested for antibodies. Three of the 57 clade 1 RNA-positive sera contained antibodies against clade 1 hepaciviruses (5.3%).

Because of previous reports of canine/equine hepaciviruses, all RT-PCR assays used in this study were also applied on specimens from horses, cats and dogs. No HCV-related sequences were found in any of the 858 canine or feline specimens. In seven of 210 horse sera (3.3%), sequences closely related to those equine hepaciviruses described previously from the US and New Zealand [24] were detected (9.5–15.0% exchanges in the 978 nucleotide NS3 gene fragment). Most of those nucleotide differences represented synonymous mutations, resulting in low amino acid distances of 0–1.2%. The novel hepaciviruses from German horses clustered phylogenetically with the previously described equine viruses (**Figure 3A**).

## Discussion

Here we found molecular evidence for viruses related to HCV in rodents. Rodent hepaciviruses were detected in four-striped grass mice from South Africa, as well as in bank voles from Central Europe. The latter have already been successfully bred under laboratory conditions, indicating an approach to establish surrogate models for hepacivirus infection [53], [54], [55], [56].

All discovered viruses originated from deep nodes close to the bifurcations separating genera within the flavivirus tree. In phylogenies on whole genome and individual gene alignments, the novel viruses clustered in a monophyletic clade with previously known hepaciviruses and GBV-B. The clade is highly diversified with NS5b amino acid sequence distances between taxa ranging up to 66.1%, exceeding that in the well-studied genus *Flavivirus* (55.8%). Maximal distances within the genera *Pegivirus* (52.9%) and *Pestivirus* (42.0%) are even lower, suggesting a particularly high diversity to exist in a tentative genus defined by the novel clade. Whereas this indicates that some or all of the novel rodent viruses together with GBV-B might alternatively form an independent genus, recent descriptions of novel pegi- and pestiviruses in bats and swine suggest the diversity also within these genera to be understudied [48], [57], [58].

Including the novel rodent viruses congeneric with HCV and canine/equine viruses, the minimal distance between the genera *Hepacivirus* and *Pegivirus* would be 73.5%. While this is lower than the 85–88% between other pairs of genera, it is consistent with a separation threshold of 72.2% between all members of the genus *Flavivirus* and Tamana bat virus, for which a separate genus has been proposed [59]. This also corresponds to inter-generic distances within other well-studied families of plus-strand RNA viruses such as the *Picornaviridae*, whose twelve genera are mostly separated by 70–80% in the *RdRp*-encoding 3D gene [60]. The genomic organization of the novel viruses provides additional criteria for tentative classification. Like all hepaciviruses and in contrast to all members of the genus *Pegivirus*, the novel viruses have a discernible *core* gene [61]. In contrast to the genus *Pestivirus* [62], their genomes contained no putative N^pro^ and E^rns^ genes in any reading frame. Finally, in contrast to the genus *Flavivirus* [63], all rodent viruses showed IRES secondary structures in their 5′-genome termini. Some of the novel rodent IRES structures appeared to contain both elements related to type 3 IRES known from hepaci- and pestiviruses and type 4 IRES known from pegiviruses. Additionally, both *M. glareolus* rodent hepacivirus polyprotein clades were preceded by predominantly pegivirus-related IRES structures, while the *R. pumilio* hepacivirus clade was preceded by a predominantly hepacivirus-related IRES. This may be compatible with ancient recombination events between *Flaviviridae* genera, a phenomenon known in the family *Picornaviridae* [64], [65].

The genetic elements potentially homologous to HCV detected in rodent viruses also included microRNA-122 binding sites in the 5′-ncr, an X-tail-like element in the 3′-terminus and a putative F gene in an alternative open reading frame (ORF) of the *R. pumilio*–associated virus. The F protein appears to be unessential for HCV replication, but the evolutionary conservation of its ORF suggests that it may play a critical regulatory role in virus propagation and survival [50]. In this regard, the F protein may be considered a counter-defensive security protein that evolved to overcome mechanisms of host resistance [66]. The absence of paramount features typical of other genera and the presence of hepacivirus-like features suggest a tentative classification of the novel rodent viruses within the genus *Hepacivirus*, rather than a novel genus.

Within the genus, phylogeny suggests early divergence of ancestral rodent viruses from a lineage leading up to HCV and canine/equine hepaciviruses. Weakness of resolution in deep bifurcations of the NS3 gene phylogeny and lack of any highly significant preference for deep topological hypotheses in the NS5B gene phylogeny underline the ancestral origin of these viruses. Within the current dataset we can consider them equidistant from HCV and the canine/equine hepaciviruses, suggesting existence of independent taxonomic entities. HCV is one viral species whose genotypes are separated by more than 30% genomic nucleotide distance [67], which corresponds to about 22–31% AA distance. Different species within the related sister genus *Pegivirus*, such as GBV-C and GBV-A, are separated from each other by about 45% AA distance [68]. Within the Genus *Flavivirus*, well-defined species such as dengue virus 1, West Nile virus, yellow fever virus and tick-borne encephalitis virus are separated from each other by 48–60% AA distance. Comparing these values we could putatively assume that both *Myodes*-associated clades distant from each other by 70% AA sequence, as well as the *Rhabdomys*-associated clade separated from both of the aforementioned by 66–69% AA sequence, might form three distinct species. The canine/equine hepacivirus clade separated from HCV by 52–53% AA sequence would then also form a separate species. Furthermore, all rodent hepacivirus clades and specifically *M. glareolus* hepacivirus clade 2 were slightly more related to GBV-B than to HCV. GBV-B causes hepatitis in experimentally infected New World primates but not in humans and chimpanzees [61]. The true host of this virus is unknown, but our findings suggest that GBV-B might originate from rodents. Nevertheless, the genetic distance of GBV-B even to its closest relative, the *Myodes* hepacivirus clade 2 (63% AA sequence), suggests GBV-B to remain a solitary representative of a separate species of hepaciviruses.

In the canine/equine clade (also termed non-primate hepaciviruses or NPHV [23]), it is striking that almost identical viruses have been found in horses and dogs. Additionally, horses but not dogs had antibodies against those viruses [24]. In the present study we augmented the number of studied dogs and horses considerably, and investigated cats in addition as these are related in the order of carnivores and have shared domestic habitats with dogs over a long history. The complete absence of viruses in cats and dogs, and the confirmation of highly similar viruses in other geographic regions, here and in another recent study [69], suggest an actual equine association of the canine/equine clade. Whether acquisition of viruses might have occurred during the domestication of horses, or whether a more generic viral association with the equine stem lineage may exist, could be clarified by testing non-domestic equids such as wild asses or zebras. However, the overall phylogenetic position and monophyly of equine viruses suggest no role as ancestral hepacivirus hosts for horses. While the rodent hepaciviruses greatly extended the genetic diversity of the genus *Hepacivirus*, their role in the evolution of HCV precursors, if any, remains to be determined.

Our serological evidence for hepaciviruses in bats is noteworthy even in absence of direct virus findings. Viruses from all *Flaviviridae* genera including *Pegivirus*, *Pestivirus* and *Flavivirus* have already been found in bats [48], [70], [71]. We could not exclude that the antibodies in bat sera reacting with HCV antigens were directed against viruses from other *Flaviviridae* genera, rather than bat hepaciviruses. However, there was no cross-reactivity between the NS3 proteins of the more closely related canine/equine hepaciviruses and HCV [24]. Similarly, the two bank vole hepacivirus clades from our study showed no serologic cross-reactivity. These data can therefore serve as very initial suggestions for the existence of bat hepaciviruses only. It should be noted that the degree of genomic similarity necessary for serologic cross-reactivity should have permitted RNA detection by the broadly reactive PCR assays used in this study. Whether bat hepaciviruses indeed exist will therefore require further evidence. A first step to this direction may be an analysis of an expanded bat sample by using the methods presented here.

Additional to phylogeny and genomic properties, the novel viruses resemble HCV in important traits of the natural history of infection. The detection of non-identical virus sequences in natural groups of animals, in combination with specific antiviral antibodies, proves continuous transmission of virus among animals. Induction of controlled infections in housed animals should thus be feasible. We found clear *in-vivo* evidence for hepatic tropism by demonstrating histopathological signs of liver inflammation, excessive viral RNA concentrations in the liver, as well as *in-situ* hybridizations demonstrating intracellular genome replication in liver cells of bank voles. A somewhat lower degree of hepatic inflammation compared to that in some HCV-infected humans might be due to the shorter life span of bank voles rarely exceeding 1–2 years in the wild, or due to a higher capacity of tissue regeneration [72], [73]. Interestingly, our serological investigations suggested bank voles might be able to clear hepacivirus infections, as antibodies did not co-occur with RNA in most, but not all animals [1]. Bank voles may therefore be more capable of clearing hepacivirus infection than humans. This would be compatible with infection patterns also observed in other *Flaviviridae* members, exemplified by the flavivirus West Nile virus in rhesus macaques, the pestivirus BVDV1 in cattle and the hepacivirus GBV-B in experimentally infected tamarins [61], [74], [75]. However, it would differ from equine hepaciviruses, in which RNA and antibodies co-occurred [24]. Controlled infection experiments in bank voles might yield relevant scenarios for the study of HCV persistence. Bank voles can be kept in the laboratory with comparatively little effort and have been used for virus infection studies, e.g., with herpesviruses, bornaviruses, hantaviruses, and flaviviruses [53], [54], [55], [56]. Efforts to establish bank vole infection models may benefit from the discovery of two highly divergent clades in this species. Knowledge of three full genomes in total should enable efficient rescue of virus from cDNA. Notably, the *Rhabdomys*-associated virus clade has a host in even closer relationship (on subfamily level) to *Mus musculus* commonly kept in laboratories, for which powerful technologies such as gene knock out and *in-vivo* imaging exist. Also for this virus clade, two different full genomes have been determined. In the present study focusing on viral ecology, however, we have not conducted infection or virus rescue trials in cell cultures or animals. Due to the strict liver tropism those viruses can be expected to be as difficult to cultivate as HCV, and we currently lack any possibilities to generate primary *Myodes* hepatocytes. The housing of those animals is in preparation, as are attempts to rescue fully sequenced viruses by reverse genetics. Additionally, our finding of presence of hepaciviruses in the *Murinae* subfamily have triggered more targeted ecological investigations to potentially identify viruses from hosts in even closer relationship to *Mus musculus*. The availability of rodent surrogate models of HCV infection may obviate one of the most critical obstacles to HCV vaccine development by obviating the need for primate experiments in early stages of experimentation [12], [34].

## Supporting Information

Figure S1 **BEAST polyprotein phylogeny including the novel rodent hepaciviruses.** The complete polyprotein sequence of all hepaciviruses was analyzed in BEAST [37] using the FLU amino acid substitution matrix and a strict clock over 10,000,000 trees sampled every 1,000 generations. After exclusion of 2,500 trees as burn-in, all trees are depicted using Densitree [39]. Blue color corresponds to most probable topologies, red to second-best, green to third-best and dark green to remaining topologies. 6,950 of 7,500 trees replicates (92.7%) yielded a monophyletic origin of the rodent hepacivirus/GBV-B clade. *M. glareolus* hepacivirus clade 1 clustered with HCV in 24 of 7,500 trees (0.3%). Hepaciviruses included were SAR46 (GenBank, KC411807) and SAR3 (KC411806) from *Rhabdomys pumilio*, RMU10-3382 (KC411777), NLR-365 (KC411796) and NLR-AP70 (KC411784) from *Myodes glareolus*, HCV-1a (NC_004102), HCV-2a (AB047639), HCV3a (X76918), HCV-4a (Y11604), HCV-5a (Y13184), HCV-6a (AY859526) and HCV-7 (EF108306), Canine/Equine hepaciviruses CHCV (JF744991), NPHV-NZP-1 (JQ434001), NPHV-A6-006 (JQ434003), NPHV-G5-077 (JQ434006), NPHV-B10-022 (JQ434004), NPHV-H10-094 (JQ434007), NPHV-G1-073 (JQ434002), NPHV-H3-011 (JQ434008), NPHV-F8-068 (JQ434005) and GBV-B (NC_001655).
(TIF)

Figure S2 **5′-non-coding genome region (5′-ncr) of European and African rodent hepaciviruses.** A. 5′-end of RMU10-3382 (GenBank, KC411777). This structure was mostly related to the *Pegivirus* type 4 IRES. Nucleotides conserved with other pegiviruses are marked in red, paired compensatory substitutions in NLR-365 (KC411796) and the partially available NLR-AP70 5-UTR (KC411784) that support the structure are in green. The Ia loop is very similar in length and shape to HCV and GBV-B. The start codon is boxed in red, additional non-functional start codons between the poly-pyrimidine stretch typical for pegiviruses and the true start codon are boxed in blue. The binding site for microRNA-122 is underlined. B. 5′-end of SAR-46 (KC411807). This structure was mostly related to a *Hepacivirus* type 3 IRES. Nucleotides conserved with HCV are marked in red. The slippery site is underlined. The start codon is boxed. Stem-loop structures in both foldings are numbered according to *Pegi*- and *Hepacivirus* reference strains.
(TIF)

Figure S3 **3′-non-coding genome region (3′-ncr) of European and African rodent hepaciviruses.** A. RMU10-3382 (GenBank, KC411777) 3′-end secondary structure. B. SAR-46 (KC411807) 3′-end secondary structure. For comparison, stem-loop (SL) SL3 of HCV1a strain H77 (NC_004102) is depicted to the right and structural similarities are highlighted in grey. PK = Pseudoknot.
(TIF)

Figure S4 **5′- and 3′-non-coding genome region (3′-ncr) of GBV-C1 and 3′-ncr of GBV-B.** A. 5′-end secondary structure of GBV-C1/HPgV, GenBank accession no. U36380. Nucleotides conserved with other pegiviruses are marked in red, paired compensatory substitutions that support the structure are in green. Stem-loop structures are numbered by order of appearance. B. 3′-end secondary structure of GBV-C1/HPgV, GenBank accession no. U36380. C. Secondary structure of the third HCV-like domain of GBV-B, GenBank accession no. AF179612. Due to the single available sequence, the remaining 3′-ncr could not be reliably folded despite repeated attempts. The nucleotide sequence immediately following the polyprotein stop codon and directly before the stem-loop structure towards the 3′-end of GBV-B is shown.
(TIF)

Figure S5 **Hepacivirus RNA concentrations in individual solid organ specimens and blood.** A. Hepacivirus-positive Myodes glareolus sampled 2008–2010 in The Netherlands and Germany. Virus concentrations are given in Log10 RNA copies per gram of tissue scaled on the y-axis for each rodent organ tested (x-axis). Horizontal bars represent mean virus concentrations per organ category. The number of available specimens per organ category is indicated below. Colors represent viruses from individual rodents as identified in the legend. B. Viral load in Log10 RNA copies per mL of blood in the same 21 animals. For one animal, no blood was available.
(TIF)

Figure S6 **In-situ hybridisation of** ***M. glareolus*** **hepacivirus clade 1 RNA in liver tissue.** A. RNA-negative M. glareolus specimen RMU10-3187 and B. RNA-positive M. glareolus specimen RMU10-3379 (viral RNA concentration, 9.4×10^8^ copies per gram, GenBank accession no. KC411778) were stained at identical conditions. Power of magnification is indicated on the left. Scale bars are depicted to the lower right corner of every panel.
(TIF)

Table S1 **Sample characteristics.** ^a^GAB = Gabon, GER = Germany, NAM = Namibia, NEL = The Netherlands, RSA = Republic of South Africa, THA = Thailand, MEX = Mexico.
(DOC)

Table S2 **Oligonucleotides used for** ***Hepacivirus*** **RT-PCR screening, genome sequencing and virus quantification.** ^a^ID = identity. ^b^numbered after CHV polyprotein (GenBank# JF744991); ^c^numbered after HCV genotype 1a polyprotein (GenBank# NC_004102);^d^numbered after CHV genome (GenBank# JF744991) ^e^R = G/A, Y = C/T, S = G/C, W = A/T, M = A/C, K = G/T, H = A/C/T, B = C/G/T, I = inosine, FAM = 6-Carboxy-Fluorescein, JOE = 2,7-Dimethoxy-4,5-dichloro-6-carboxyfluorescein, VIC = proprietary dye (Life Technologies, Darmstadt, Germany), BHQ = Black hole quencher, MGBNFQ = Minor groove binder Non fluorescent quencher; ^f^+t/+c = Locked nucleic acids (LNA) First round RT-PCR used the SuperScript III (SSIII) one-step RT-PCR kit (Invitrogen, Karlsruhe, Germany) with 5 µL of RNA, 400 nM each of 1st-round primers or an equimolar mix of primers, 1 µg bovine serum albumin, 0.2 mM of each dNTP and 2.4 mM of MgSO4. Second round 50 µL Platinum Taq (Invitrogen) reactions used 1 µL of 1st-round PCR product, 2.5 mM MgCl2 and 400 nM each of 2nd-round primers. First round RT-PCR reactions were used a touchdown protocol with reverse transcription at 48° for 30 minutes, denaturation at 95° for 3 minutes, followed by PCR 10 cycles of 15 sec at 94°C, 20 sec at 60°C with a decrease of 1°C per cycle, and extension at 72°C for 45 seconds, followed by another 40 cycles at 50°C annealing temperature. Second round reactions used the same cycling protocol without the RT step. RNA quantification was performed in 25 µL reaction volumes using the SSIII One-Step RT-PCR system (Invitrogen) as described above with 300 nmol/L of respective forward and reverse primers and 200 nmol/L of respective probes. Amplification involved 15 min at 55°C; 3 min at 95°C; 45 cycles of 15 sec at 94°C, and 25 sec at 58°C. Fluorescence was measured at the 58°C annealing/extension step. Published assays from which oligonucleotide primers were used in this study included [35], [79], [80], [81], [82].
(DOC)

Table S3 **Putative cleavage sites for cellular signal peptidases within the N-terminal half of hepacivirus polyproteins.** NN: neural networks; HMM: hidden Markov models (the values represent probabilities for putative SP cleavage sites). Only SP cleavage sites predicted by both NN and HMM were considered. All scores were re-calculated upon putting a suggested cleavage site at amino acid position 20 of a query polypeptide. *Y-scores were zero for these sites, however they were supported by uncorrected S-scores (not shown). Hepaciviruses included were SAR46 (KC411807) and SAR3 (KC411806) from *Rhabdomys pumilio*, RMU10-3382 (KC411777), NLR-365 (KC411796) and NLR-AP70 (KC411784) from *Myodes glareolus*, HCV-1a (NC_004102) and GBV-B (NC_001655).
(DOC)

Table S4 **Minimum amino acid identity of the novel rodent to prototype hepaciviruses.** [1] *Rhabdomys pumilio* clade 1 hepacivirus: SAR46 (KC411807); SAR3 (KC411806) [2] *Myodes glareolus* clade 1 hepacivirus: RMU10-3382 (KC411777); NLR-365, KC411796 [3] *Myodes glareolus* clade 2 hepacivirus: NLR-AP70 (KC411784) HCV: HCV-1a (NC_004102), HCV-2a (AB047639), HCV3a (X76918), HCV-4a (Y11604), HCV-5a (Y13184), HCV-6a (AY859526) and HCV-7 (EF108306); Canine/Equine hepaciviruses CHV (JF744991), NPHV-NZP-1 (JQ434001), NPHV-A6-006 (JQ434003), NPHV-G5-077 (JQ434006), NPHV-B10-022 (JQ434004), NPHV-H10-094 (JQ434007), NPHV-G1-073 (JQ434002), NPHV-H3-011 (JQ434008), NPHV-F8-068 (JQ434005); GBV-B (NC_001655) In italics: Highest identity of any hepacivirus with HCV in matrix (canine/equine clade in all genes). Underlined: Highest identity of any hepacivirus with GBV-B in matrix (a rodent clade in all genes). In bold type: Smallest identity value in matrix.
(DOC)
