# Supplementary figures and images for: Evidence for Novel Hepaciviruses in Rodents
Source: PLoS Pathog. 2013 Jun 20;9(6):e1003438. doi: 10.1371/journal.ppat.1003438 (PMC3688547; doi:10.1371/journal.ppat.1003438)

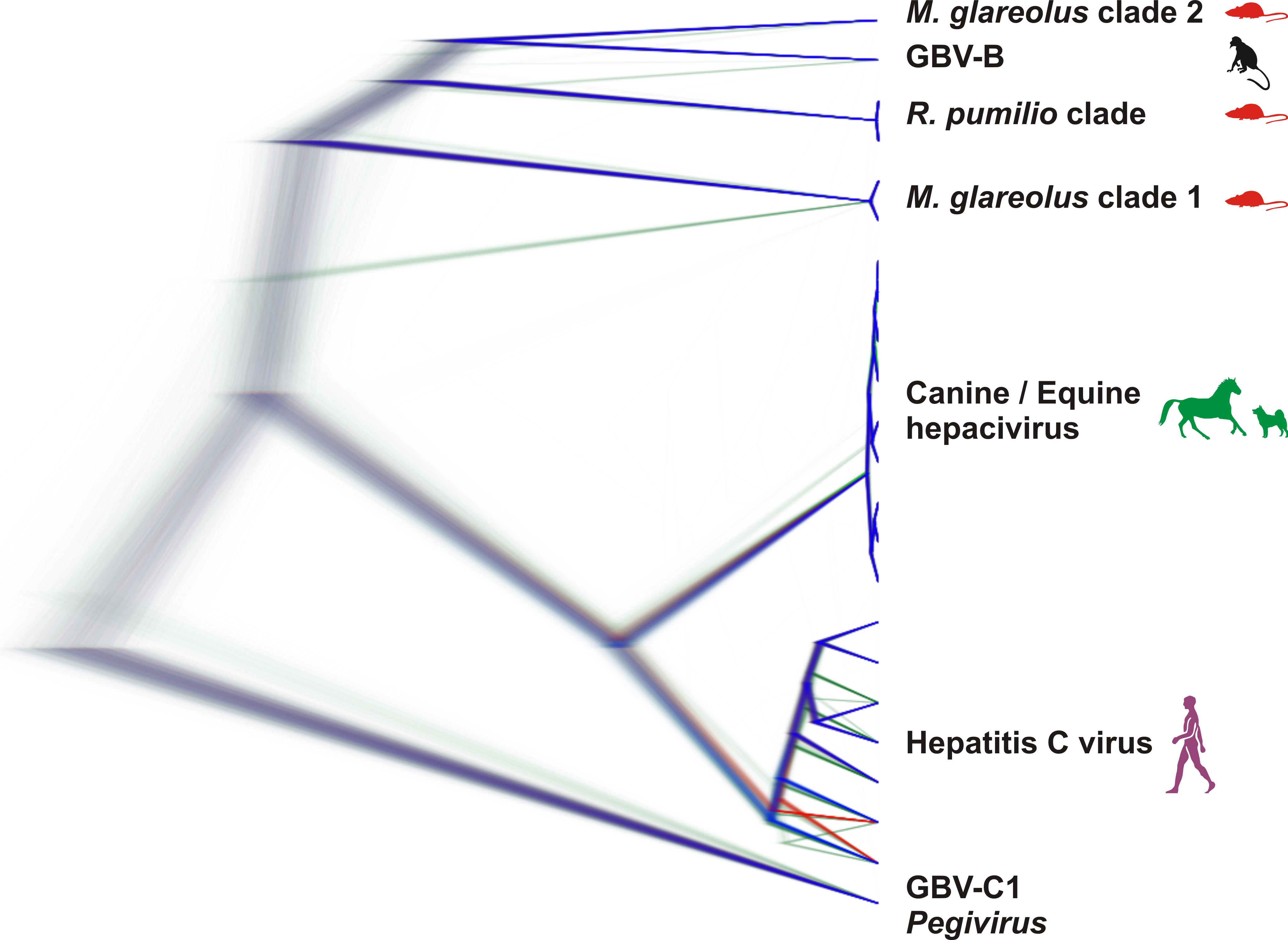

Supplement: Figure S1 — BEAST polyprotein phylogeny including the novel rodent hepaciviruses. The complete polyprotein sequence of all hepaciviruses was analyzed in BEAST [37] using the FLU amino acid substitution matrix and a strict clock over 10,000,000 trees sampled every 1,000 generations. After exclusion of 2,500 trees as burn-in, all trees are depicted using Densitree [39]. Blue color corresponds to most probable topologies, red to second-best, green to third-best and dark green to remaining topologies. 6,950 of 7,500 trees replicates (92.7%) yielded a monophyletic origin of the rodent hepacivirus/GBV-B clade. M. glareolus hepacivirus clade 1 clustered with HCV in 24 of 7,500 trees (0.3%). Hepaciviruses included were SAR46 (GenBank, KC411807) and SAR3 (KC411806) from Rhabdomys pumilio, RMU10-3382 (KC411777), NLR-365 (KC411796) and NLR-AP70 (KC411784) from Myodes glareolus, HCV-1a (NC_004102), HCV-2a (AB047639), HCV3a (X76918), HCV-4a (Y11604), HCV-5a (Y13184), HCV-6a (AY859526) and HCV-7 (EF108306), Canine/Equine hepaciviruses CHCV (JF744991), NPHV-NZP-1 (JQ434001), NPHV-A6-006 (JQ434003), NPHV-G5-077 (JQ434006), NPHV-B10-022 (JQ434004), NPHV-H10-094 (JQ434007), NPHV-G1-073 (JQ434002), NPHV-H3-011 (JQ434008), NPHV-F8-068 (JQ434005) and GBV-B (NC_001655). (TIF) [file ppat.1003438.s001.tif]

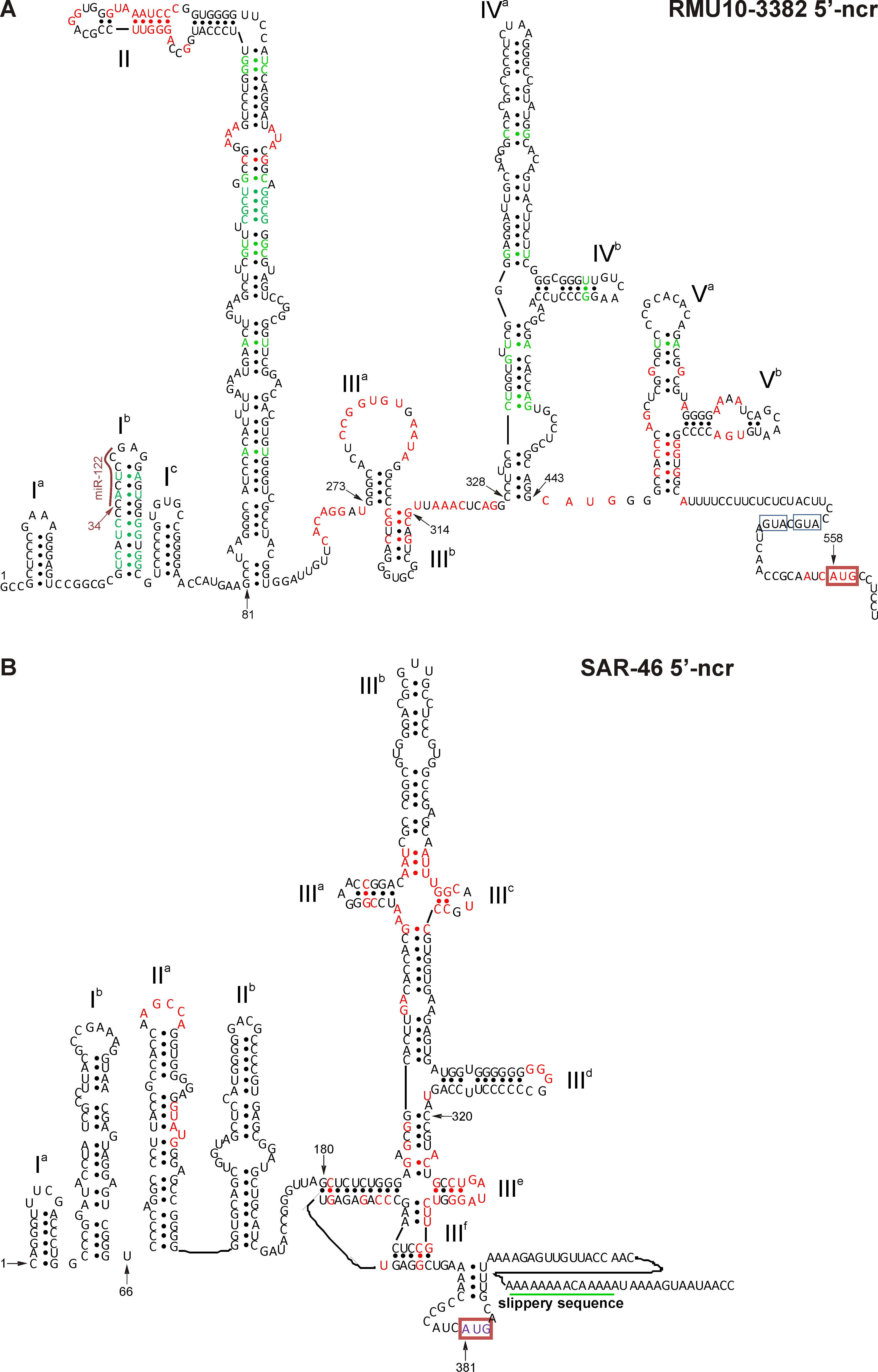

Supplement: Figure S2 — 5′-non-coding genome region (5′-ncr) of European and African rodent hepaciviruses. A. 5′-end of RMU10-3382 (GenBank, KC411777). This structure was mostly related to the Pegivirus type 4 IRES. Nucleotides conserved with other pegiviruses are marked in red, paired compensatory substitutions in NLR-365 (KC411796) and the partially available NLR-AP70 5-UTR (KC411784) that support the structure are in green. The Ia loop is very similar in length and shape to HCV and GBV-B. The start codon is boxed in red, additional non-functional start codons between the poly-pyrimidine stretch typical for pegiviruses and the true start codon are boxed in blue. The binding site for microRNA-122 is underlined. B. 5′-end of SAR-46 (KC411807). This structure was mostly related to a Hepacivirus type 3 IRES. Nucleotides conserved with HCV are marked in red. The slippery site is underlined. The start codon is boxed. Stem-loop structures in both foldings are numbered according to Pegi- and Hepacivirus reference strains. (TIF) [file ppat.1003438.s002.tif]

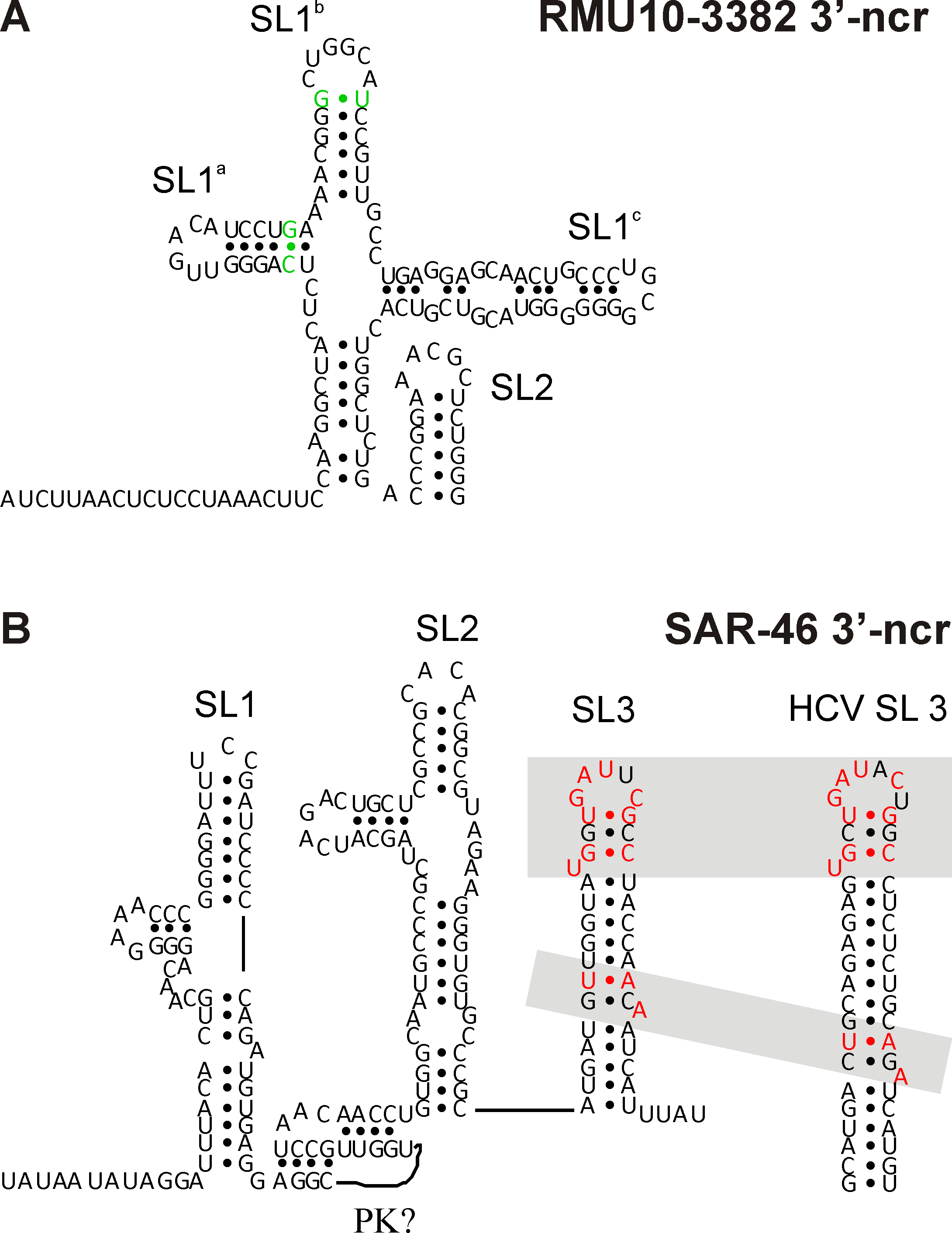

Supplement: Figure S3 — 3′-non-coding genome region (3′-ncr) of European and African rodent hepaciviruses. A. RMU10-3382 (GenBank, KC411777) 3′-end secondary structure. B. SAR-46 (KC411807) 3′-end secondary structure. For comparison, stem-loop (SL) SL3 of HCV1a strain H77 (NC_004102) is depicted to the right and structural similarities are highlighted in grey. PK = Pseudoknot. (TIF) [file ppat.1003438.s003.tif]

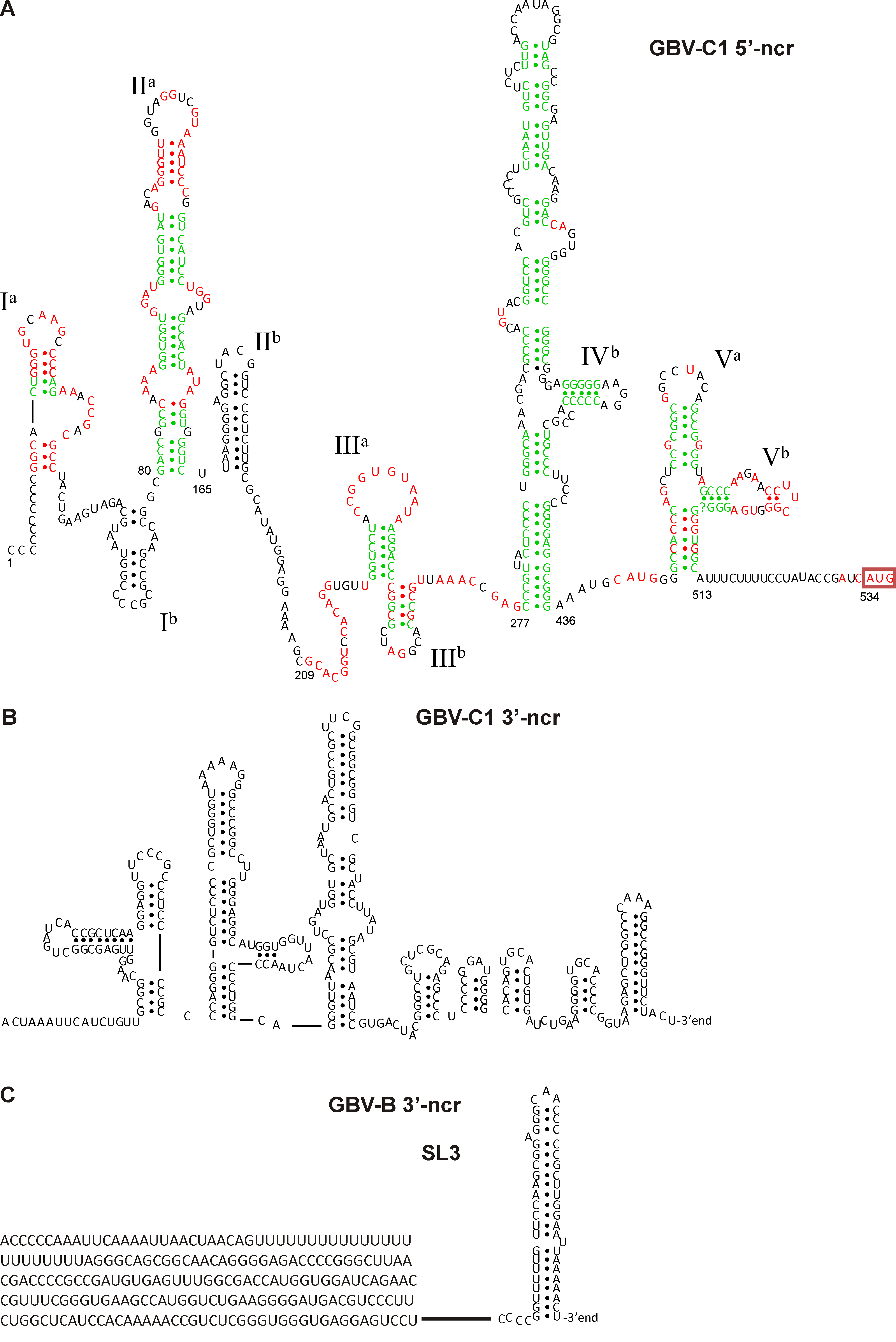

Supplement: Figure S4 — 5′- and 3′-non-coding genome region (3′-ncr) of GBV-C1 and 3′-ncr of GBV-B. A. 5′-end secondary structure of GBV-C1/HPgV, GenBank accession no. U36380. Nucleotides conserved with other pegiviruses are marked in red, paired compensatory substitutions that support the structure are in green. Stem-loop structures are numbered by order of appearance. B. 3′-end secondary structure of GBV-C1/HPgV, GenBank accession no. U36380. C. Secondary structure of the third HCV-like domain of GBV-B, GenBank accession no. AF179612. Due to the single available sequence, the remaining 3′-ncr could not be reliably folded despite repeated attempts. The nucleotide sequence immediately following the polyprotein stop codon and directly before the stem-loop structure towards the 3′-end of GBV-B is shown. (TIF) [file ppat.1003438.s004.tif]

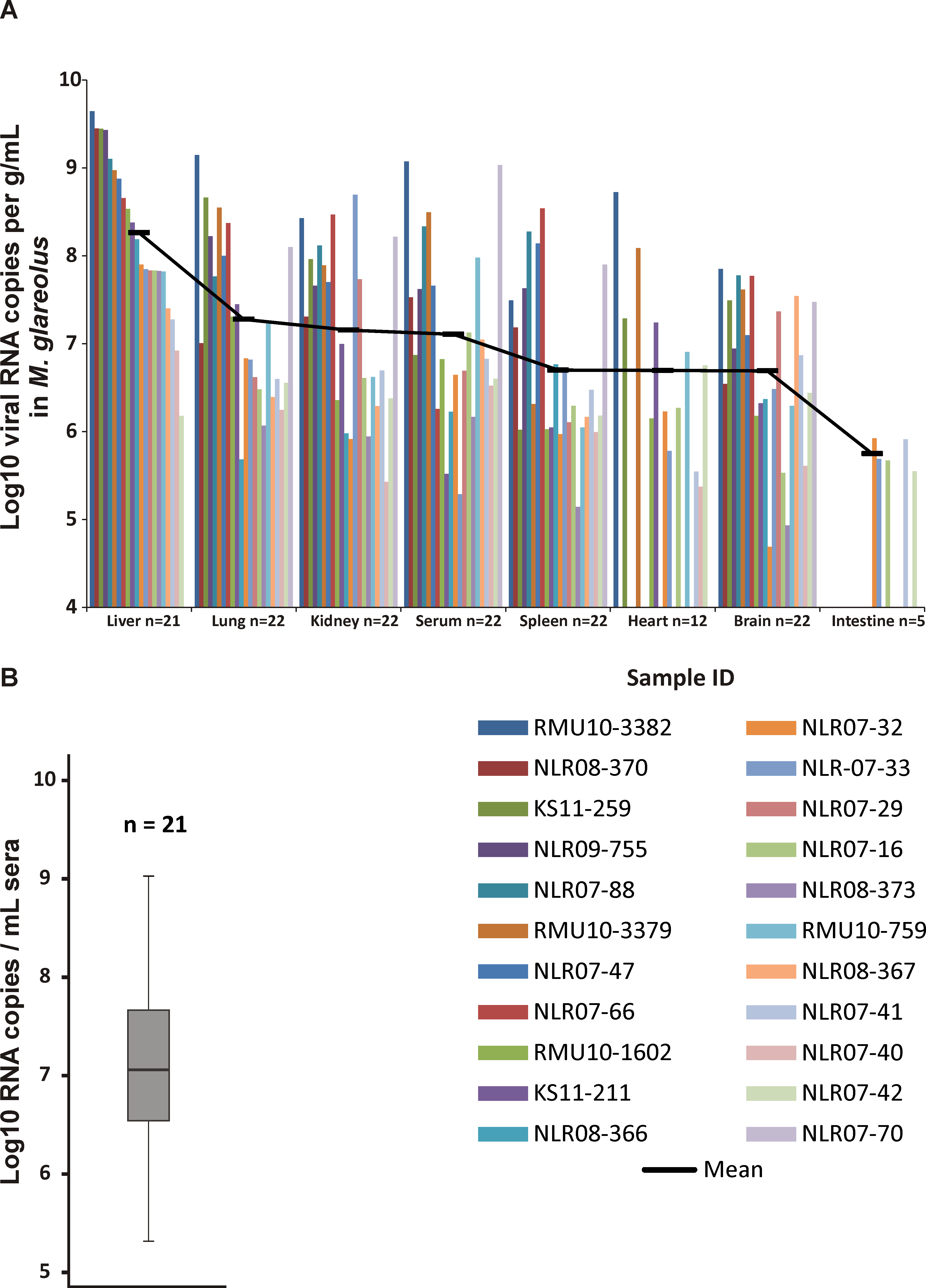

Supplement: Figure S5 — Hepacivirus RNA concentrations in individual solid organ specimens and blood. A. Hepacivirus-positive Myodes glareolus sampled 2008–2010 in The Netherlands and Germany. Virus concentrations are given in Log10 RNA copies per gram of tissue scaled on the y-axis for each rodent organ tested (x-axis). Horizontal bars represent mean virus concentrations per organ category. The number of available specimens per organ category is indicated below. Colors represent viruses from individual rodents as identified in the legend. B. Viral load in Log10 RNA copies per mL of blood in the same 21 animals. For one animal, no blood was available. (TIF) [file ppat.1003438.s005.tif]

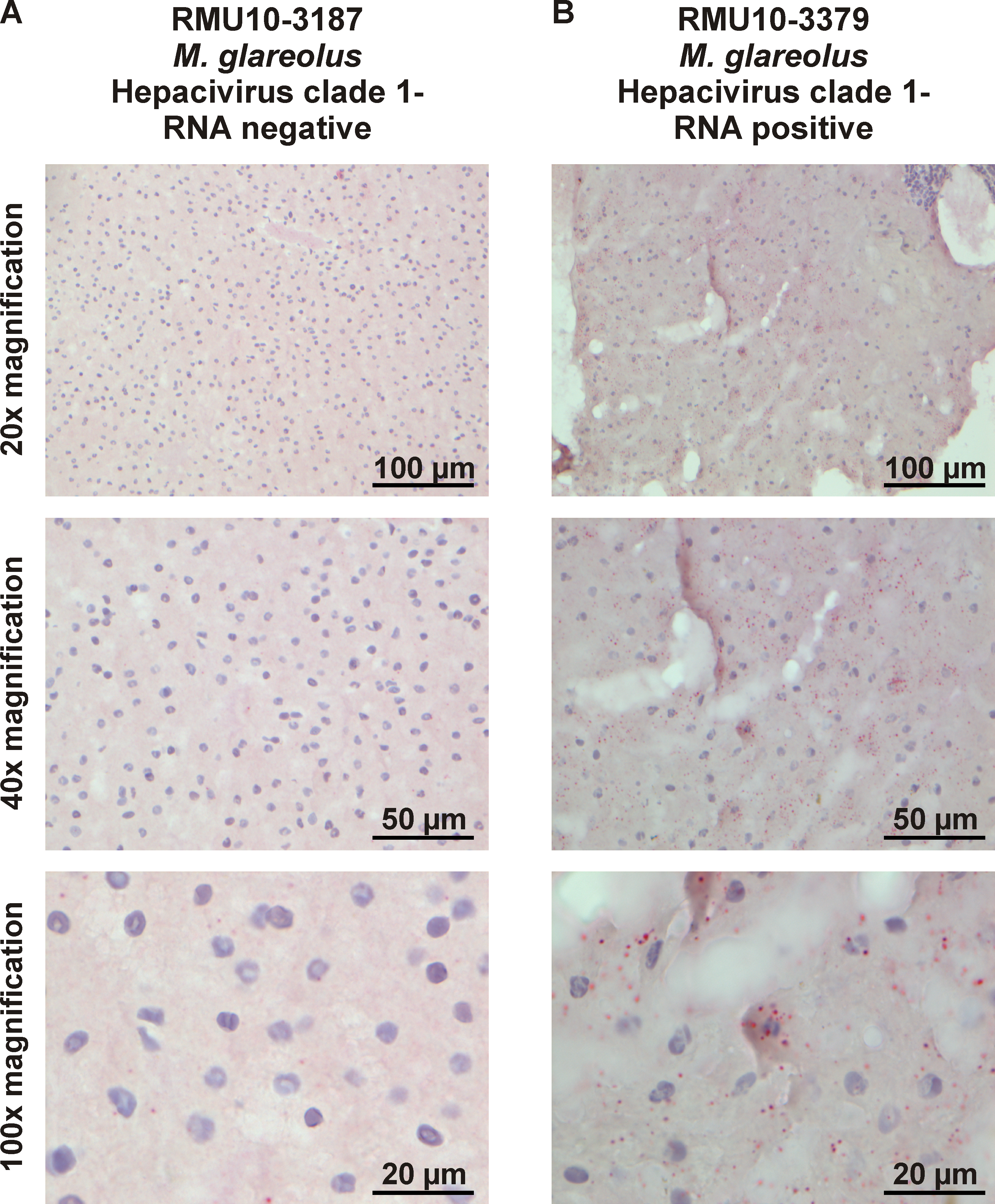

Supplement: Figure S6 — In-situ hybridisation of M. glareolus hepacivirus clade 1 RNA in liver tissue. A. RNA-negative M. glareolus specimen RMU10-3187 and B. RNA-positive M. glareolus specimen RMU10-3379 (viral RNA concentration, 9.4×10^8^ copies per gram, GenBank accession no. KC411778) were stained at identical conditions. Power of magnification is indicated on the left. Scale bars are depicted to the lower right corner of every panel. (TIF) [file ppat.1003438.s006.tif]
