# Supplementary material for: Evidence for Novel Hepaciviruses in Rodents
Source: PLoS Pathog. 2013 Jun 20;9(6):e1003438. doi: 10.1371/journal.ppat.1003438 (PMC3688547; doi:10.1371/journal.ppat.1003438)
Supplement: Table S1 — Sample characteristics. aGAB = Gabon, GER = Germany, NAM = Namibia, NEL = The Netherlands, RSA = Republic of South Africa, THA = Thailand, MEX = Mexico. (DOC) [file ppat.1003438.s007.doc]

Supplementary Table S1. Sample characteristics

| Order | Family | Species | No. of samples | No. PCR positive (% positive) | Specimen | Sampling site^a^ (year) |
| --- | --- | --- | --- | --- | --- | --- |
| Chiroptera | Emballonuridae | *Coleura afra* | 14 |  | blood | GAB (2009) |
|  | Emballonuridae | *Saccopteryx bilineata* | 14 |  | blood | PAN (2010,2011) |
|  | Emballonuridae | *Saccopteryx leptura* | 3 |  | blood | PAN (2010,2011) |
|  | Hipposideridae | *Hipposideros cf. ruber* | 51 |  | blood | GAB (2009) |
|  | Hipposideridae | *Hipposideros gigas* | 129 |  | blood | GAB (2009) |
|  | Molossidae | *Molossus molossus* | 1 |  | blood | PAN (2010,2011) |
|  | Mormoopidae | *Pteronotus parnellii* | 53 |  | blood | PAN (2010,2011) |
|  | Natalidae | *Natalus stramineus* | 1 |  | blood | PAN (2010,2011) |
|  | Noctilionidae | *Noctilio leporinus* | 11 |  | blood | PAN (2005,2010,2011) |
|  | Phyllostomidae | *Artibeus jamaicensis* | 981 |  | blood | PAN (2005,2010,2011) |
|  | Phyllostomidae | *Artibeus lituratus* | 94 |  | blood | PAN (2005,2010,2011) |
|  | Phyllostomidae | *Artibeus phaeotis* | 10 |  | blood | PAN (2010,2011) |
|  | Phyllostomidae | *Artibeus watsoni* | 16 |  | blood | PAN (2010,2011) |
|  | Phyllostomidae | *Carollia castanea* | 30 |  | blood | PAN (2010,2011) |
|  | Phyllostomidae | *Carollia perspicillata* | 20 |  | blood | PAN (2010,2011) |
|  | Phyllostomidae | *Chiroderma villosum* | 2 |  | blood | PAN (2010,2011) |
|  | Phyllostomidae | *Desmodus rotundus* | 2 |  | blood | PAN (2010,2011) |
|  | Phyllostomidae | *Glossophaga sorcina* | 25 |  | blood | PAN (2010,2011) |
|  | Phyllostomidae | *Lophostoma brasiliense* | 4 |  | blood | PAN (2010,2011) |
|  | Phyllostomidae | *Lophostoma silvicolum* | 17 |  | blood | PAN (2010,2011) |
|  | Phyllostomidae | *Micronycteris hirsuta* | 3 |  | blood | PAN (2010,2011) |
|  | Phyllostomidae | *Micronycteris microtis* | 9 |  | blood | PAN (2010,2011) |
|  | Phyllostomidae | *Micronycteris minuta* | 1 |  | blood | PAN (2010,2011) |
|  | Phyllostomidae | *Mimon crenulatum* | 4 |  | blood | PAN (2010,2011) |
|  | Phyllostomidae | *Phylloderma stenops* | 5 |  | blood | PAN (2010,2011) |
|  | Phyllostomidae | *Phyllostomus discolor* | 10 |  | blood | PAN (2010,2011) |
|  | Phyllostomidae | *Phyllostomus hastatus* | 16 |  | blood | PAN (2010,2011) |
|  | Phyllostomidae | *Platyrrhinus helleri* | 6 |  | blood | PAN (2010,2011) |
|  | Phyllostomidae | *Tonatia saurophila* | 14 |  | blood | PAN (2010,2011) |
|  | Phyllostomidae | *Trachops cirrhosus* | 17 |  | blood | PAN (2010,2011) |
|  | Phyllostomidae | *Uroderma bilobatum* | 54 |  | blood | PAN (2010,2011) |
|  | Phyllostomidae | *Vampyressa pusilla* | 3 |  | blood | PAN (2010,2011) |
|  | Phyllostomidae | *Vampyrodes caraccioli* | 4 |  | blood | PAN (2010,2011) |
|  | Pteropodidae | *Dobsonia praedatrix* | 9 |  | blood | PNG (2002) |
|  | Pteropodidae | *Eidolon helvum* | 348 |  | blood | GHA (2009,2010) |
|  | Pteropodidae | *Epomops franquetti* | 100 |  | blood | GAB (2009) |
|  | Pteropodidae | *Hysignathus monstrosus* | 100 |  | blood | GAB (2009) |
|  | Pteropodidae | *Melonycteris melanops* | 7 |  | blood | PNG (2002) |
|  | Pteropodidae | *Micropterus pusillus* | 100 |  | blood | GAB (2009) |
|  | Pteropodidae | *Myonycteris torquata* | 100 |  | blood | GAB (2009) |
|  | Pteropodidae | *Pteropus alecto* | 3 |  | blood | AUS (2006) |
|  | Pteropodidae | *Pteropus lylei* | 200 |  | blood | THA (2008) |
|  | Pteropodidae | *Pteropus poliocephalus* | 24 |  | blood | AUS (2006) |
|  | Pteropodidae | *Rousettus aegyptiacus* | 204 |  | blood | GAB (2009) |
|  | Pteropodidae | *Rousettus amplexicaudatus* | 1 |  | blood | PNG (2002) |
|  | Rhinolophidae | *Rhinolophus cf. alcyone* | 16 |  | blood | GAB (2009) |
|  | Thyropteridae | *Thyroptera tricolor* | 3 |  | blood | PAN (2010,2011) |
|  | Vespertilionidae | *Miniopterus inflatus* | 51 |  | blood | GAB (2009) |
|  | Vespertilionidae | *Myotis daubentonii* | 42 |  | blood | GER (2009) |
|  | Vespertilionidae | *Myotis nigricans* | 5 |  | blood | PAN (2010,2011) |
|  | Vespertilionidae | *Rhogeessa tumida* | 2 |  | blood | PAN (2010,2011) |
|  | **Subtotal** | **51 species** | ***2939*** |  |  |  |
| Rodentia | Cricetidae | *Arvicola amphibius* | 3 |  | blood | GER (2007-2011) |
|  | Cricetidae | *Baiomys sp.* | 51 |  | liver | MEX (2011-2012) |
|  | Cricetidae | *Microtus agrestis* | 125 |  | blood | GER (2007-2011) |
|  | Cricetidae | *Microtus arvalis* | 352 |  | blood | GER (2007-2011), NEL (2007-2008) |
|  | Cricetidae | *Microtus sp.* | 37 |  | blood | GER (2007-2011) |
|  | Cricetidae | *Myodes glareolus* | 1465 | 27 (1.8) | blood | GER (2007-2011), NEL (2007-2010) |
|  | Cricetidae | *Oryzomys couesi* | 22 |  | liver | MEX (2011-2012) |
|  | Cricetidae | *Oryzomys sp.* | 63 |  | liver | MEX (2011-2012) |
|  | Cricetidae | *Osgoodomys sp.* | 51 |  | liver | MEX (2011-2012) |
|  | Cricetidae | *Peromyscus sp.* | 2 |  | liver | MEX (2011-2012) |
|  | Cricetidae | *Reithrodontomys sp.* | 22 |  | liver | MEX (2011-2012) |
|  | Cricetidae | *Sigmodon sp.* | 49 |  | liver | MEX (2011-2012) |
|  | Heteromyidae | *Liomys sp.* | 89 |  | liver | MEX (2011-2012) |
|  | Muridae | *Aethomys namaquensis* | 6 |  | blood | RSA (2008) |
|  | Muridae | *Apodemus agrarius* | 98 |  | blood | GER (2007-2011) |
|  | Muridae | *Apodemus flavicollis* | 701 |  | blood | GER (2007-2011) |
|  | Muridae | *Apodemus spp.* | 47 |  | blood | GER (2007-2011) |
|  | Muridae | *Apodemus sylvaticus* | 338 |  | blood | GER (2007-2011), NEL (2007-2010) |
|  | Muridae | *Bandicota indica* | 151 |  | blood | THA (1995-2007) |
|  | Muridae | *Bandicota savilei* | 9 |  | blood | THA (1995-2005) |
|  | Muridae | *Bandicota sp.* | 1 |  | blood | THA (1995-2005) |
|  | Muridae | *Desmodillus sp.* | 1 |  | blood | RSA (2008) |
|  | Muridae | *Heimyscus fumosus* | 1 |  | blood | GAB (2011) |
|  | Muridae | *Hybomys univittatus* | 1 |  | blood | GAB (2011) |
|  | Muridae | *Lemniscomys striatus* | 18 |  | blood | GAB (2011) |
|  | Muridae | *Lophuromys sp.* | 1 |  | blood | GAB (2011) |
|  | Muridae | *Malacomys longipes* | 1 |  | blood | GAB (2011) |
|  | Muridae | *Mastomys sp.* | 1 |  | blood | RSA (2008) |
|  | Muridae | *Micromys minutus* | 4 |  | blood | GER (2007-2011) |
|  | Muridae | *Mus musculus* | 32 |  | blood/liver | GER (2007-2011), GAB (2011), MEX (2011-2012) |
|  | Muridae | *Nannomys setulosus* | 7 |  | blood | GAB (2011) |
|  | Muridae | *Otomys sp.* | 21 |  | blood | RSA (2008) |
|  | Muridae | *Parotomys littledaly* | 1 |  | blood | RSA (2008) |
|  | Muridae | *Parotomys sp.* | 28 |  | blood | RSA (2008) |
|  | Muridae | *Praomys misonnei* | 25 |  | blood | GAB (2011) |
|  | Muridae | *Rattus argentiventer* | 28 |  | blood | THA11995-2005) |
|  | Muridae | *Rattus exulans* | 2 |  | blood | THA (2005) |
|  | Muridae | *Rattus losea* | 7 |  | blood | THA (1995) |
|  | Muridae | *Rattus norvegicus* | 174 |  | blood | GER (2007-2011),THA (1995-1996), NEL (2009-2010) |
|  | Muridae | *Rattus rattus* | 168 |  | blood | THA (1995-2007), NEL (2009-2010), RSA (2008), GAB (2011) |
|  | Muridae | *Rattus sp.* | 5 |  | blood | GER (2007-2011) |
|  | Muridae | *Rattus tiomanicus* | 27 |  | blood | THA (1996-2007) |
|  | Muridae | *Rhabdomys pumilio* | 518 | 10 (1.9) | blood | RSA (2008), NAM (2008) |
|  | Nesomyidae | *Cricetomys gambianus* | 1 |  | blood | GAB (2011) |
|  | Nesomyidae | *Petromyscus sp.* | 2 |  | blood | RSA (2008) |
|  | Sciuridae | *Sciurus vulgaris* | 12 |  | blood | GER (2007-2011) |
|  | Myocastoridae | *Myocastor coypus* | 2 |  | blood | NEL (2009) |
|  | **Subtotal** | **41 species** | ***4770*** |  |  |  |
| Carnivora | Canidae | *Canis lupus familiaris* | 239 |  | nasal swab | GER (2010-2011) |
|  | Canidae | *Canis lupus familiaris* | 167 |  | blood | GER (2012-2013) |
|  | Felidae | *Felis silvestris catus* | 452 |  | nasal swab | GER (2010-2011) |
|  | **Subtotal** | **2 species** | *858* |  |  |  |
| Perissodactyla | Equidae | *Equus ferus caballus* | 210 | 7 (3.3) | blood | GER (2012) |
|  | **Subtotal** | **1 species** | *210* |  |  |  |
|  | Total | 95 species | 8777 | 44 (0.5) |  |  |
