# Supplementary material for: Evidence for Novel Hepaciviruses in Rodents
Source: PLoS Pathog. 2013 Jun 20;9(6):e1003438. doi: 10.1371/journal.ppat.1003438 (PMC3688547; doi:10.1371/journal.ppat.1003438)
Supplement: Table S2 — Oligonucleotides used for Hepacivirus RT-PCR screening, genome sequencing and virus quantification. aID = identity. bnumbered after CHV polyprotein (GenBank# JF744991); cnumbered after HCV genotype 1a polyprotein (GenBank# NC_004102);dnumbered after CHV genome (GenBank# JF744991) eR = G/A, Y = C/T, S = G/C, W = A/T, M = A/C, K = G/T, H = A/C/T, B = C/G/T, I = inosine, FAM = 6-Carboxy-Fluorescein, JOE = 2,7-Dimethoxy-4,5-dichloro-6-carboxyfluorescein, VIC = proprietary dye (Life Technologies, Darmstadt, Germany), BHQ = Black hole quencher, MGBNFQ = Minor groove binder Non fluorescent quencher; f+t/+c = Locked nucleic acids (LNA) First round RT-PCR used the SuperScript III (SSIII) one-step RT-PCR kit (Invitrogen, Karlsruhe, Germany) with 5 µL of RNA, 400 nM each of 1st-round primers or an equimolar mix of primers, 1 µg bovine serum albumin, 0.2 mM of each dNTP and 2.4 mM of MgSO4. Second round 50 µL Platinum Taq (Invitrogen) reactions used 1 µL of 1st-round PCR product, 2.5 mM MgCl2 and 400 nM each of 2nd-round primers. First round RT-PCR reactions were used a touchdown protocol with reverse transcription at 48° for 30 minutes, denaturation at 95° for 3 minutes, followed by PCR 10 cycles of 15 sec at 94°C, 20 sec at 60°C with a decrease of 1°C per cycle, and extension at 72°C for 45 seconds, followed by another 40 cycles at 50°C annealing temperature. Second round reactions used the same cycling protocol without the RT step. RNA quantification was performed in 25 µL reaction volumes using the SSIII One-Step RT-PCR system (Invitrogen) as described above with 300 nmol/L of respective forward and reverse primers and 200 nmol/L of respective probes. Amplification involved 15 min at 55°C; 3 min at 95°C; 45 cycles of 15 sec at 94°C, and 25 sec at 58°C. Fluorescence was measured at the 58°C annealing/extension step. Published assays from which oligonucleotide primers were used in this study included [35], [79], [80], [81], [82]. (DOC) [file ppat.1003438.s008.doc]

**Supplementary Table S2. Oligonucleotides used for *Hepacivirus*** RT-PCR screening, genome sequencing and virus quantification

| **Genomic target region** | **Oligonucleotide ID^a^** | **Sequence (5’-3’)^e^** | **Polarity** | **Assay usage** |
| --- | --- | --- | --- | --- |
| NS3 version A/Bb | Hepaci-F3628 | GCICCIACIGGIAGYGGIAA | + | Screening hemi-nested RT-PCR A/B 1st round |
|  | Hepaci-F3868a | TAYGAYGTIATIATITGYGAYGARTG | + | Screening hemi-nested RT-PCR A/B 2nd round |
|  | Hepaci-F3868b | TAYGAYGTIATIATITGYGAYGA | + | Screening hemi-nested RT-PCR A/B 2nd round |
|  | Hepaci-F3979 | GCIACIGCIACICCICCIGG | + | Screening hemi-nested RT-PCR A/B 2nd round |
|  | Hepaci-R4268 | CCIGTCATIAGRGCRTCIGT | - | Screening hemi-nested RT-PCR B 1st and 2nd round |
|  | Hepaci-R4302 | CARTCIRTIACIGARTCRAARTYICC | - | Screening hemi-nested RT-PCR A 1st and 2nd round |
|  | GBV-B-NS5BF496 | GAAATGAGATGTGTTGAGAAGATG | + | Screening hemi-nested RT-PCR 1st round |
| GBV-B NS5B | GBV-B-NS5BF502 | AGATGTGTTGAGAAGATGTACTACGGT | + | Screening hemi-nested RT-PCR 2nd round |
|  | GBV-B-NS5BR686 | TCCTCGGGTGTGATGGTACTGTCAAAACA | - | Screening hemi-nested RT-PCR 1st and 2nd round |
|  | murHep-1b-F | AACRACGCCCCATCCYAAC | + |  |
| NS3 | murHep-1-Ra | GCCGYTTCCCGTAGAATTGC | - | *M. glareolus* hepacivirus clade 1 screening real time RT-PCR |
|  | murHep-1-FAM | FAM-ACTGAGGTTGAGCTGGGCTCGAGT-BHQ1 | + |  |
| HCV 5’UTR | 1CH | As in [79] |  | Screening nested RT-PCR |
|  | 2CH |  |  |  |
|  | 4CH |  |  |  |
|  | 1TS |  |  |  |
|  | DM100 |  |  |  |
| HCV NS5B | DM101 | As in [80,81] |  | Screening hemi-nested RT-PCR |
|  | PR3 |  |  |  |
| HCV X-tail | F5 | As in [35] |  | Screening RT-PCR |
|  | xtR2 |  |  |  |
| Genus *Flavivirus* NS5 | PF1S |  |  |  |
|  | PF2R-bis | As in [83] |  | Screening hemi-nested RT-PCR |
|  | PF3S |  |  |  |
| Genus *Pestivirus* NS5 | PestiV-NS5-F | GCIATICCIAAGAATGAGAAGAG | + |  |
|  | PestiV-NS5-R | AAGCCRTCRTCICCRCASACGTG | - | Screening hemi-nested RT-PCR |
|  | PestiV-NS5-Rnest | TCIGGTTGICCGCTICCTCTTTG | - |  |
| NS5B version Ac | HCV1a-F7820 | TGGGIIIIKCITAYGGITTYCARTAYWCICC | + | Hemi-nested RT-PCR 1st round for genome islet |
|  | HCV1a-F7906 | GGITTITCITAYGAYACIIGITGYTTYGAY | + | Hemi-nested RT-PCR 2^nd^ round for genome islet |
|  | HCV1a-R8237a | CTITCICAIATIACGRIIARRTCRTCICCRCA | - | Alternative hemi-nested RT-PCR 1^st^ and 2^nd^ round for genome islet |
|  | HCV1a-R8237b | CTITCICAIATIACGRIIARRTCRTCICC | - |  |
|  | HCV1a-R8378a | GCIACIGAIACRTTIGAKGARCA | - | Hemi-nested RT-PCR 1^st^ and 2^nd^ round for genome islet |
| NS5B version B^c^ | HCV1a-7900 | CCIATGGSITTYGCITGTGAYAC | + | Hemi-nested RT-PCR 1^st^ round for genome islet |
|  | HCV1a-F7921 | ACAATTTGYTTYGAYTCIACYGTIAC | + | Hemi-nested RT-PCR 2^nd^ round for genome islet |
|  | HCV1a-R8378b | GCIGARCTIACRTTISTKGARCAIGA | - | NS5 Hemi-nested RT-PCR 1^st^ and 2^nd^ round for genome islet |
| Core^b^ | HCV-F361 | TGGGGMCIIMIIGAYCCYCG | + | Hemi-nested RT-PCR 1^st^ round for genome islet |
|  | HCV-F376 | CCYCGSSRIMRITCICGIAAYITIGG | + | Hemi-nested RT-PCR 2^nd^ round for genome islet |
|  | HCV-R542a | CCWGTIRYIIARTTIRYICCRTCYTC | - | Hemi-nested RT-PCR 1^st^ and 2^nd^ round for genome islet |
| Core^b^ | rodHCV-F365 | GGCSITCIGACCCIAGICGIAGITC | + | Hemi-nested RT-PCR 1^st^ round for genome islet |
|  | rodHCV-F386 | GGTCIAGIAAYGTIGGICAITTIGTIGA | + | Hemi-nested RT-PCR 2^nd^ round for genome islet |
|  | rodHCV-R542b | CTIGTIACIATRTTIAYIATRTCYTC | - | Hemi-nested RT-PCR 1^st^ and 2^nd^ round for genome islet |
| 5’-UTR^d^ | HCV-F150 | GSWSCYYCYAGGICCMCCCC | + | Hemi-nested RT-PCR 1^st^ and 2^nd^ round for genome islet |
|  | HCV-R371 | CTCRTGIISYAIGGTCTACRAGRCC | - | Hemi-nested RT-PCR 1^st^ round for genome islet |
|  | HCV-R342 | GGIGCICTCGCAAGCRYGCCYATCAG | - | Hemi-nested RT-PCR 2^nd^ round for genome islet |
| Core-NS3 | HCV-F361mod | TGGGGMCIIMIIGAYCCYCGSSRIMRITCICGIAAYITIGG | + | 5’-extension of NS3 fragments using stringent reverse transcription, virus-specific inner reverse primers and non-stringent 1^st^ round PCR conditions |
|  | HCV-F515 | TIGAGGAYGGIGYIAAYTIIGYIACWGG | + |  |
|  | HCV-F538 | ACWGGIAAYITICCYGGIIIIIIYTTYTCTATYTTY | + |  |
|  | HCV1a-F1741 | TGYCCYACIGAYTGYTTYMG | + |  |
|  | HCV1a-F2836 | GGIACITWYITITATGAICAYYT | + |  |
|  | HCV1a-F2836mod | YGGIACYTWYITITAYGAYCAY | + |  |
|  | HCV1a-F2977 | TGYGGIGAYATTITIIGIGGIYTICCIGT | + |  |
|  | HCV1a-F3493 | TCIGGIGGICCICTITTGTG | + |  |
| NS3-NS5b | HCV-R4922^c^ | CCARGCRTAIGCIIIGTCRWAGSMCTC | - | 3’-extension of NS3/NS5b fragments using non-stringent reverse transcription and virus-specific inner forward primers |
|  | HCV-R5072^c^ | CAYTCY+t+cYATY+t+cRTC^g^ | - |  |
|  | HCV-R5549^c^ | GGSIIIWCICCIIICAWGATYTTRAA | - |  |
|  | HCV-R7001^c^ | YTCICCYTCIAGIGGIGGCAT | - |  |
|  | HCV-R9101^c^ | YTCCAIGCYCGIAAIGGIGG | - |  |
|  | HCV-R9361^c^ | GTTAGIAGGACAGAIACIACRAGIAG | - |  |
|  | rodHCV-NS3extRa | GGRTCAGGRTGTAARCATTG | - |  |
|  | rodHCV-NS3extRb | CGCGCTGCTTCATGATCTC | - |  |
| NS3 | rodHCV-EB-rtF | ATGGGCCCCTACATGCAA | + | One-step real time RT-PCR-based virus-specific quantification |
|  | rodHCV-EB-rtP | FAM-AGGCGTACAACATCGCGCCCA-BHQ1 | + |  |
|  | rodHCV-EB-rtR | TCGATGTCTCACCCGTGTACA | - |  |
|  | rodHCV-S-rtF | CGTCGAAGCTGGTATGTGACA | + |  |
|  | rodHCV-S-rtP | JOE-CTTGGCCAGCCTACTGCGGCA-BHQ1 | + |  |
|  | rodHCV-S-rtR | AGTACGCAACGGCAGTGATG | - |  |
|  | rodHCV-LK-rtF | TGTGACACCTTAGCCAGTTTGC | + |  |
|  | rodHCV-LK-rtP | FAM-ACGGCAGCACGGCATCACTGC-BHQ1 | + |  |
|  | rodHCV-LK-rtR | GCTCGCCTCGGTAGTAAGCA | - |  |
|  | rodHCV-LL-rtF | TGTATACGGGCGAGACATCAAT | + |  |
|  | rodHCV-LL-rtP | JOE-CACGGGCACCAAATTGACATATGCC-BHQ1 | + |  |
|  | rodHCV-LL-rtR | CATGGCTGCGGCTTTCC | - |  |
|  | rodHCV-NJ-rtF | AGCATGGGCCCCTATATGC | + |  |
|  | rodHCV-NJ-rtP | FAM-AAAGGCGTATAACATTGCGCCCAGC-BHQ1 | + |  |
|  | rodHCV-NJ-rtR | ATTGATGTCTCGCCCGTATACA | - |  |
|  | rodHCV-NS2-rtF | GTCAAGGGCAGGTGGATTGT | + |  |
|  | rodHCV-NS2-rtP | FAM-TTTGACACCCTGCGGACTGGCA-BHQ1 | + |  |
|  | rodHCV-NS2-rtR | TGGAAGGCATGAAAATCAAGTG | - |  |
|  | rodHCV-NS26-rtF | CCCGGGATGTGCAGTGA | + |  |
|  | rodHCV-NS26-rtP | JOE-CCCCCACCCAAATGTAGATGAAACTCCC-BHQ1 | + |  |
|  | rodHCV-NS26-rtR | AGGTCGCCCTCTTCAGGAA | - |  |
|  | rodHCV-NLRBA-rtF | CTTTTGCGCGTCCAAGCT | + |  |
|  | rodHCV-NLRBA-rtP | JOE-TGTGACACCCTCGCCAGCTTGCT-BHQ1 | + |  |
|  | rodHCV-NLRBA-rtR | GGCAGTGATGCCGTGTTGT | - |  |
|  | rodHCV-NLR-AP-rtF | CAGCCAAATGCAAACTTGTCA | + |  |
|  | rodHCV-NLR-AP-rtP | FAM-TTAGCCACTGCCACCCCACCTGGTA-BHQ1 | + |  |
|  | rodHCV-NLR-AP-rtR | GTTGGAGTGAGGTTGCATTGAA | - |  |
|  | rodHCV-NLR-AY-rtF | CATTTTGGCCACGGCTACTC | + |  |
|  | rodHCV-NLR-AY-rtP | JOE-TCCCGGGTGTTCCACAACGCC-BHQ1 | + |  |
|  | rodHCV-NLR-AY-rtR | GCTCCACCTCCGTGATATTAGG | - |  |
|  | rodHCV-NLR-BA-rtF | CTTTTGCGCGTCCAAGCT | + |  |
|  | rodHCV-NLR-BA-rtP | FAM-TGTGACACCCTCGCCAGCTTGCT-BHQ1 | + |  |
|  | rodHCV-NLR-BA-rtR | GGCAGTGATGCCGTGTTGT | - |  |
|  | rodHCV-NLR-BJ-rtF | TGGGACCGGCACTAAGTTG | + |  |
|  | rodHCV-NLR-BJ-rtP | JOE-CATACGCGACCTACGGCAAGGCA-BHQ1 | + |  |
|  | rodHCV-NLR-BJ-rtR | GATAGCAAACTCGTGTCCATAGCA | - |  |
|  | rodHCV-NLR-G-rtF | AAGCATGGGCCCGTAYATG | + |  |
|  | rodHCV-NLR-G-rtP | FAM-AAAAGGCGTACAACATCGCGCCAA-BHQ1 | + |  |
|  | rodHCV-NLR-G-rtR | TGGATGTTTCACCCGTATACACA | - |  |
|  | rodHCV-NLR-L-N-P-rtF | CATTTTGGCCACGGCTACAC | + |  |
|  | rodHCV-NLR-L-N-P-rtP | JOE-TGTTCCACCACACCCCACCCAAAC-BHQ1 | + |  |
|  | rodHCV-NLR-L-N-P-rtR | CTAGACCCTAGCTCCACCTCAGTT | - |  |
|  | rodHCVeur-rtF3 | GAGGTGCARTTCTAYGGGAA | + |  |
|  | rodHCVeur-rtPmgb | VIC-CAYCTCATCTTYTGYGCRTC-MGBNFQ | + |  |
|  | rodHCVeur-rtR | GGGTGTCRCAYACCWGCTT | - |  |
