# Supplementary material for: Evidence for Novel Hepaciviruses in Rodents
Source: PLoS Pathog. 2013 Jun 20;9(6):e1003438. doi: 10.1371/journal.ppat.1003438 (PMC3688547; doi:10.1371/journal.ppat.1003438)
Supplement: Table S3 — Putative cleavage sites for cellular signal peptidases within the N-terminal half of hepacivirus polyproteins. NN: neural networks; HMM: hidden Markov models (the values represent probabilities for putative SP cleavage sites). Only SP cleavage sites predicted by both NN and HMM were considered. All scores were re-calculated upon putting a suggested cleavage site at amino acid position 20 of a query polypeptide. *Y-scores were zero for these sites, however they were supported by uncorrected S-scores (not shown). Hepaciviruses included were SAR46 (KC411807) and SAR3 (KC411806) from Rhabdomys pumilio, RMU10-3382 (KC411777), NLR-365 (KC411796) and NLR-AP70 (KC411784) from Myodes glareolus, HCV-1a (NC_004102) and GBV-B (NC_001655). (DOC) [file ppat.1003438.s009.doc]

**Supplementary Table S3**. Putative cleavage sites for cellular signal peptidases within the N-terminal half of hepacivirus polyproteins

| **Virus** | **Predicted SPase cleavage site** | **NN, Y score** | **HMM** |
| --- | --- | --- | --- |
| **HCV-1a** | ASA_191_|YQ | 0.871 | 0.897 |
| **HCV-1a** | VDA_383_|ET | 0.907 | 0.959 |
| **HCV-1a** | AEA_746_|AL | 0.670 | 0.914 |
| **HCV-1a** | AYA_809_|LD | 0.850 | 0.994 |
| **HCV-1a** | VEA_855_|QL | 0.805 | 0.889 |
| **GBV-B** | CSG_156_|AR | 0.866 | 0.611 |
| **GBV-B** | TSG_349_|NP | 0.858 | 0.866 |
| **GBV-B** | ASG_613_|YP | 0.777 | 0.848 |
| **GBV-B** | AAA_681_|QP | 0.652 | 0.813 |
| **GBV-B** | ASA_732_|FD | 0.857 | 0.980 |
| **RMU10-3382** | AVS_163_|HW | 0.604 | 0.644 |
| **RMU10-3382** | AEG_348_|LP | 0.831 | 0.935 |
| **RMU10-3382** | VRS_617_|KY | 0* | 0.636 |
| **RMU10-3382** | ANA_635_|LV | 0.710 | 0.901 |
| **RMU10-3382** | AQG_680_|GC | 0.492 | 0.675 |
| **NLR-365** | AVS_163_|HW | 0.602 | 0.644 |
| **NLR-365** | AEG_348_|LP | 0.841 | 0.936 |
| **NLR-365** | VRS_617_|KY | 0* | 0.691 |
| **NLR-365** | ADA_635_|LV | 0.714 | 0.862 |
| **NLR-365** | ARS_680_|GC | 0* | 0.579 |
| **SAR3** | VEP_172_|KP | 0.855 | 0.968 |
| **SAR3** | SVA_369_|AP | 0.761 | 0.821 |
| **SAR3** | YAK_624_|PP | 0.813 | 0.966 |
| **SAR3** | VEA_677_|FS | 0.705 | 0.931 |
| **SAR46** | VEP_172_|KP | 0.855 | 0.968 |
| **SAR46** | SVA_369_|AP | 0.761 | 0.821 |
| **SAR46** | YAK_624_|PP | 0.813 | 0.966 |
| **SAR46** | VEA_677_|FS | 0.694 | 0.931 |
| **NLR-AP70** | AVT_149_|NC | 0.676 | 0.538 |
| **NLR-AP70** | AAA_333_|AS | 0.772 | 0.609 |
| **NLR-AP70** | AFA_613_|FT | 0.792 | 0.985 |
| **NLR-AP70** | TSA_667_|YS | 0.588 | 0.767 |
