# Supplementary material for: Evidence for Novel Hepaciviruses in Rodents
Source: PLoS Pathog. 2013 Jun 20;9(6):e1003438. doi: 10.1371/journal.ppat.1003438 (PMC3688547; doi:10.1371/journal.ppat.1003438)
Supplement: Table S4 — Minimum amino acid identity of the novel rodent to prototype hepaciviruses. [1] Rhabdomys pumilio clade 1 hepacivirus: SAR46 (KC411807); SAR3 (KC411806) [2] Myodes glareolus clade 1 hepacivirus: RMU10-3382 (KC411777); NLR-365, KC411796 [3] Myodes glareolus clade 2 hepacivirus: NLR-AP70 (KC411784) HCV: HCV-1a (NC_004102), HCV-2a (AB047639), HCV3a (X76918), HCV-4a (Y11604), HCV-5a (Y13184), HCV-6a (AY859526) and HCV-7 (EF108306); Canine/Equine hepaciviruses CHV (JF744991), NPHV-NZP-1 (JQ434001), NPHV-A6-006 (JQ434003), NPHV-G5-077 (JQ434006), NPHV-B10-022 (JQ434004), NPHV-H10-094 (JQ434007), NPHV-G1-073 (JQ434002), NPHV-H3-011 (JQ434008), NPHV-F8-068 (JQ434005); GBV-B (NC_001655) In italics: Highest identity of any hepacivirus with HCV in matrix (canine/equine clade in all genes). Underlined: Highest identity of any hepacivirus with GBV-B in matrix (a rodent clade in all genes). In bold type: Smallest identity value in matrix. (DOC) [file ppat.1003438.s010.doc]

**Supplementary Table S4. Minimum amino acid identity of the novel rodent to prototype hepaciviruses**

| **Core**   |  | [1] | [2] | [3] | HCV | CHV EqHV | GBV-B | | --- | --- | --- | --- | --- | --- | --- | | [1] | 98.8 |  |  |  |  |  | | [2] | 38.4 | 100 |  |  |  |  | | [3] | 34.0 | 36.1 | 100 |  |  |  | | HCV | 31.0 | **27.6** | 31.3 | 80.6 |  |  | | CHV  EqHV | 34.5 | 35.0 | 31.5 | *48.1* | 91.2 |  | | GBV-B | 30.0 | 30.3 | 39.6 | 27.7 | 28.4 | 100 | | **E1**   |  | [1] | [2] | [3] | HCV | CHV  EqHV | GBV-B | | --- | --- | --- | --- | --- | --- | --- | | [1] | 99.5 |  |  |  |  |  | | [2] | 26.1 | 97.8 |  |  |  |  | | [3] | 40.6 | 29.5 | 100 |  |  |  | | HCV | 23.9 | **19.0** | 20.6 | 51.0 |  |  | | CHV  EqHV | 23.5 | 23.2 | 23.6 | *26.3* | 92.0 |  | | GBV-B | 33.3 | 26.6 | 36.1 | 24.1 | 24.6 | 100 | |
| --- | --- | --- | --- | --- | --- | --- | --- | --- | --- | --- | --- | --- | --- | --- | --- | --- | --- | --- | --- | --- | --- | --- | --- | --- | --- | --- | --- | --- | --- | --- | --- | --- | --- | --- | --- | --- | --- | --- | --- | --- | --- | --- | --- | --- | --- | --- | --- | --- | --- | --- | --- | --- | --- | --- | --- | --- | --- | --- | --- | --- | --- | --- | --- | --- | --- | --- | --- | --- | --- | --- | --- | --- | --- | --- | --- | --- | --- | --- | --- | --- | --- | --- | --- | --- | --- | --- | --- | --- | --- | --- | --- | --- | --- | --- | --- | --- | --- | --- | --- |
| **E2**   |  | [1] | [2] | [3] | HCV | CHV  EqHV | GBV-B | | --- | --- | --- | --- | --- | --- | --- | | [1] | 98.4 |  |  |  |  |  | | [2] | 19.4 | 95.8 |  |  |  |  | | [3] | 28.7 | 15.6 | 100 |  |  |  | | HCV | 20.4 | **15.1** | 20.3 | 66.2 |  |  | | CHV  EqHV | 21.1 | 17.2 | 18.7 | *37.3* | 89.3 |  | | GBV-B | 34.4 | 16.9 | 28.9 | 18.2 | 16.6 | 100 | | **NS2**   |  | [1] | [2] | [3] | HCV | CHV  EqHV | GBV-B | | --- | --- | --- | --- | --- | --- | --- | | [1] | 98.1 |  |  |  |  |  | | [2] | 20.8 | 89.0 |  |  |  |  | | [3] | 20.2 | 23.2 | 100 |  |  |  | | HCV | 16.9 | 20.3 | 17.1 | 53.2 |  |  | | CHV  EqHV | **13.8** | 20.3 | 17.9 | *34.3* | 86.8 |  | | GBV-B | 18.0 | 23.0 | 27.7 | 19.8 | 19.0 | 100 | |
| **NS3**   |  | [1] | [2] | [3] | HCV | CHV  EqHV | GBV-B | | --- | --- | --- | --- | --- | --- | --- | | [1] | 99.4 |  |  |  |  |  | | [2] | 39.3 | 97.3 |  |  |  |  | | [3] | 42.8 | 42.4 | 100 |  |  |  | | HCV | 38.6 | 42.2 | **37.9** | 78.6 |  |  | | CHV  EqHV | 40.1 | 41.9 | 40.0 | *57.5* | 98.9 |  | | GBV-B | 40.5 | 42.0 | 45.5 | 39.3 | 41.7 | 100 | | **NS4**   |  | [1] | [2] | [3] | HCV | CHV  EqHV | GBV-B | | --- | --- | --- | --- | --- | --- | --- | | [1] | 100 |  |  |  |  |  | | [2] | 29.3 | 97.8 |  |  |  |  | | [3] | 27.1 | 28.8 | 100 |  |  |  | | HCV | 27.5 | 26.7 | 24.7 | 64.1 |  |  | | CHV  EqHV | 26.5 | **24.4** | 27.0 | *44.1* | 96.5 |  | | GBV-B | 30.1 | 29.7 | 33.6 | 25.8 | 26.8 | 100 | |
| **NS5A**   |  | [1] | [2] | [3] | HCV | CHV  EqHV | GBV-B | | --- | --- | --- | --- | --- | --- | --- | | [1] | 99.5 |  |  |  |  |  | | [2] | **15.4** | 87.3 |  |  |  |  | | [3] | 20.8 | 16.3 | 100 |  |  |  | | HCV | 19.9 | 16.3 | 20.7 | 57.5 |  |  | | CHV  EqHV | 22.5 | 16.8 | 20.6 | *38.5* | 89.9 |  | | GBV-B | 21.3 | 15.9 | 23.1 | 21.5 | 19.6 | 100 | | **NS5B**   |  | [1] | [2] | [3] | HCV | CHV  EqHV | GBV-B | | --- | --- | --- | --- | --- | --- | --- | | [1] | 99.5 |  |  |  |  |  | | [2] | 41.4 | 98.2 |  |  |  |  | | [3] | 44.6 | 38.6 | 100 |  |  |  | | HCV | 34.3 | 36.3 | **34.1** | 71.1 |  |  | | CHV  EqHV | 35.5 | 38.3 | 35.7 | *54.7* | 94.2 |  | | GBV-B | 46.0 | 41.5 | 47.0 | 35.6 | 35.8 | 100 | |
